# Chemical Composition and Biological Activity of Extracts Obtained by Supercritical Extraction and Ethanolic Extraction of Brown, Green and Red Propolis Derived from Different Geographic Regions in Brazil

**DOI:** 10.1371/journal.pone.0145954

**Published:** 2016-01-08

**Authors:** Bruna Aparecida Souza Machado, Rejane Pina Dantas Silva, Gabriele de Abreu Barreto, Samantha Serra Costa, Danielle Figuerêdo da Silva, Hugo Neves Brandão, José Luiz Carneiro da Rocha, Odir Antônio Dellagostin, João Antônio Pegas Henriques, Marcelo Andres Umsza-Guez, Francine Ferreira Padilha

**Affiliations:** 1 Institute of Research and Technology, Tiradentes University, Aracaju, Sergipe, Brazil; 2 Faculty of Technology, SENAI/CIMATEC, National Service of Industrial Learning–SENAI, Salvador, Bahia, Brazil; 3 Faculty of Pharmacy, State University of Feira de Santana, Feira de Santana, Bahia, Brazil; 4 Technology Development Center, Federal University of Pelotas, Pelotas, Rio Grande do Sul, Brazil; 5 Biotechnology, University of Caixias do Sul, Caixias do Sul, Rio Grande do Sul, Brazil; 6 Biotechnology, Federal University of Bahia, Salvador, Bahia, Brazil; College of Agricultural Sciences, UNITED STATES

## Abstract

The variations in the chemical composition, and consequently, on the biological activity of the propolis, are associated with its type and geographic origin. Considering this fact, this study evaluated propolis extracts obtained by supercritical extraction (SCO_2_) and ethanolic extraction (EtOH), in eight samples of different types of propolis (red, green and brown), collected from different regions in Brazil. The content of phenolic compounds, flavonoids, *in vitro* antioxidant activity (DPPH and ABTS), Artepillin C, p-coumaric acid and antimicrobial activity against two bacteria were determined for all extracts. For the EtOH extracts, the anti-proliferative activity regarding the cell lines of B16F10, were also evaluated. Amongst the samples evaluated, the red propolis from the Brazilian Northeast (states of Sergipe and Alagoas) showed the higher biological potential, as well as the larger content of antioxidant compounds. The best results were shown for the extracts obtained through the conventional extraction method (EtOH). However, the highest concentrations of Artepillin C and p-coumaric acid were identified in the extracts from SCO_2_, indicating a higher selectivity for the extraction of these compounds. It was verified that the composition and biological activity of the Brazilian propolis vary significantly, depending on the type of sample and geographical area of collection.

## Introduction

Propolis is characterized as a complex and resinous mixture produced by bees (*Apis melífera*) through the collection of variable vegetable sources [1–4]. Propolis is constituted by a variety of chemical compounds, including the derivatives of cinnamic acid, such as p-coumaric acid and Artepillin C, substituted benzoic acids, phenolic acids, flavonoids and aminoacids [5–7]. Different studies have already proved that the chemical composition of the propolis, and consequently its biological effects, depends on various factors such as the geographic origin, types of vegetable sources, time of collection and season of the year [8–10]. Although propolis is considered a complex mixture, its biological activities are reported due to the presence of the flavonoids, phenolic acids and ethers in the propolis composition [11–12].

Currently, the Brazilian propolis can be classified in 13 different types, according to its physical-chemical properties and the geographic area where it was found. Park et al. [6] classified the propolis samples collected from different regions around Brazil in 12 groups, according to appearance and colour of the extracts. The *Baccharis dracunculifolia* DC (Asteraceae) [13–14], a native plant from Brazil, is the most important botanical source of propolis in the Brazilian southeast, known as green propolis. Afterwards, a new propolis was found in hives located alongside the coast and mangroves in the Brazilian northeast and it was classified as a propolis of the group 13. This propolis is called red propolis, with botanical origin from *Dalbergia ecastophyllum* (L.) Taub. (Fabaceae) [15–16].

Propolis extracts are more commonly obtained through conventional techniques, such as the ethanolic extraction, aqueous extraction or by Soxhlet [17–19]. In the last few years, different studies showed the extraction with supercritical fluid (SFE) as an important alternative method to obtain compounds derived from natural matrices [20–23], including, for example, propolis [24–26]. This process shows advantages over the conventional ones, such as higher selectivity, reduction in use of organic solvents, obtaining extracts with high biological value and use of carbon dioxide (CO_2_) as extractor solvent [27–29]. According to Machado et al. [30], when it is compared the extracts obtained by SFE with other conventional extractive methods, it is noted that the quantity of compounds obtained by SFE from the same matrix is very superior. However, despite the higher number of compounds extracted, often the yield of the extraction process is lower, which could indicate a higher selectivity.

Distinct biological properties and chemical compositions are described for the samples of propolis collected in Brazil, which is explained by the great Brazilian biodiversity. The antimicrobial and antitumoral capacity of red propolis were evidenced by many authors [18,31–32]. Different studies identified antifungal [33], immunomodulation [34–35], anti-ulcer [19,36] and anti-inflammatory [37] properties for samples of Brazilian green propolis. Fernandes et al. [38] evaluated the antioxidant effects and the (anti)genotoxicity in samples of brown propolis from the Brazilian savanna. In the study performed by Wilson et al. [39], the antimicrobial activity and chemical composition of 12 samples of propolis collected in different regions of the United States were evaluated. The profiles obtained by chromatography, as well as the activity of the microorganisms tested showed very distinct aspects, indicating that the variation of results was due to the geographical region. In view of that, the objective of this study was to perform the chemical characterization, evaluate the antioxidant capacity and antimicrobial activity of propolis extracts obtained by SFE and ethanolic extraction (conventional), as well as *in vitro* evaluation of antitumor of the ethanolic extracts against the cell lines of B16F10, from eight samples (brown, green and red) collected in different geographical regions of Brazil.

## Material and Methods

### Materials and reagents

Ethanol (HPLC degree) and aseptic acid (HPLC degree) were obtained from Merck Co. (Darmstadt, Germany) and methanol (PA) from Sigma-Aldrich Chemical Co. (St. Louis, MO, USA). A cellulose ester membrane filter of 0.45 μm (SLCR025NS, Millipore® Co., Bedford, Massachusetts, USA) was used. The carbon dioxide (CO_2_) used in the extraction had 99.9% purity (White Martins Gases Industrials–São Paulo, Brazil). The standard 3,5-diprenil-4-hidroxicinamic (Artepillin C–cas number 72944-19-5) was acquired from Wako Pure Chemical Industries, Ltd. (Osaka, Japan) and the Acid 4-hidroxicinamic (p-coumaric acid–cas number 501-98-4), 2,2-diphenyl-1-picrylhydrazyl (DPPH), Acid Gallic (cas number 149-91-7), Quercetin (cas number 117-39-5), 2,2′-azinobis-(3-ethylbenzothiazoline-6-sulfonic acid (ABTS) (cas number 30931-67-0) and (±)-6-Hydroxy-2,5,7,8-tetramethylchromane-2-carboxylic acid (Trolox) (cas number 53188-07-1) were acquired from Sigma-Aldrich Chemical Co. (St. Louis, MO, USA).

### Obtaining and processing of propolis samples

Approximately 700–1000g of propolis samples were donated by the company Apis Nativa Produtos Naturais (Prodapys–Santa Catarina–Brazil), originated from the different regions in Brazil, during the period of July to September 2013. Two samples of red propolis were from the Brazilian northeast (Alagoas and Sergipe), three samples of brown propolis from the south (Santa Catarina, Rio Grande do Sul and Parana) and three samples of green propolis were from the south and southeast of Brazil (Parana and Minas Gerais) (two samples from different regions) (Table 1). The samples of propolis were crushed in a grinder (Cadence–Brazil) and then sieved (60 mesh), in order to obtain an adequate granulometry (approximately 0.250 mm) to increase the surface area and homogenise the start material in the extraction processes. Small quantities (250g) of propolis were kept in a fridge at -10°C, in bottles protected with laminated paper in inert atmospheric conditions (N_2_) in order to avoid degradation of the material.

**Table 1:** Identification of propolis samples from different regions of Brazil and analysed in this study.

Sample Identification	State and region of Brazil	Colour Type
SER	Sergipe–Northeast	Red
RAL	Alagoas–Northeast	Red
GMG_1_	Minas Gerais–Southeast	Green
GMG_2_	Minas Gerais–Southeast	Green
GPR	Paraná –South	Green
BSC	Santa Catarina–South	Brown
BRS	Rio Grande do Sul–South	Brown
BPR	Paraná –South	Brown

### Characterization of raw material

The determinations of humidity, protein and total ash contents were made according to the official methods of Association of Official Agricultural Chemists (AOAC) [40]. The total lipids were extracted and quantified through the cold extraction method described by Bligh & Dyer [41]. The determination of the mineral content was made in a digital flame photometer (DM-62, DIGIMED, São Paulo—Brazil) and the fiber content was obtained through the automatic fibre analyser (A-220, ANKON, New York–USA) [42]. The quantification of the water activity took place using a decagon LabMaster (Novasina, Lachen–Switzerland), with electrolytic cell CM-2. The analyses were performed in triplicate. The Scanning electron microscopy (SEM) was performed in a scanning electron microscope JEOL JSM-6390LV (USA). After drying in an oven (105°C/45 min), the sample of crushed propolis was fixed manually using a tweezer (PELCO® Tweezers) of aluminum metal surfaces covered with carbon double-sided tape, called stubs. Because of the need for interaction of the electron beam with the sample, it was performed by coating deposition of metallic gold ions (sputtering). The sample was metalized in gold in a “Sputter oater” from Balzers, model SCD 50 (20nm). Then the stubs containing the metallic samples were stored in plastic boxes (storage boxes), duly sealed with parafilm (PARAFILM® M) to prevent moisture absorption. After 24 hours of rest, the samples were analyzed at different magnifications (Voltage 12 kV, Working Distance 12 mm, Spot size 44, Vacuum Mode HV).

### Obtaining propolis extracts by supercritical fluid extraction (SFE) and low pressure extraction (LPE)

#### Supercritical extracts using CO_2_ as supercritical fluid

The equipment used for obtaining the propolis extracts was the pilot unity called SFT-110 Supercritical Fluid Extractor (Supercritical Fluid Technologies, Inc.), composed by a high pressure bomb (capacity of up to 10,000 psi), extraction cell (capacity of 100 ml), furnace (containing a pre-warmer), static/dynamics valve and restrictor valve, flow meter, flux totalizer (ITRÓN, ACD G1.0, Argentina) and CO_2_ cylinder. A CO_2_ cylinder with fishing tube was used to ensure that only CO_2_ in liquid state was used in the system, a requirement of the SFT-110. The CO_2_ was not re-used in the system.

**Fig 1:**
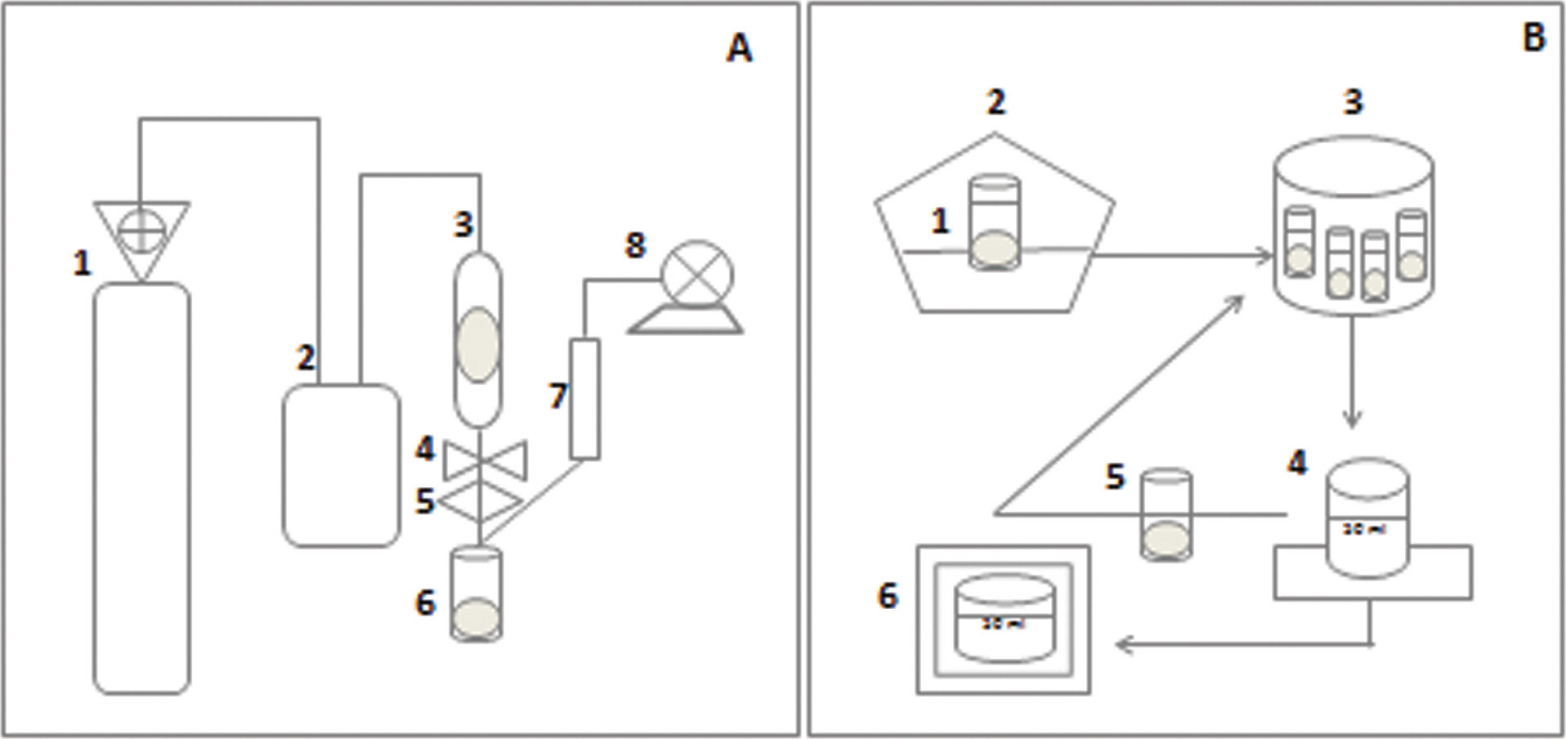
Extraction Process Stages. (A) High pressure extraction (SFE) 1 –CO_2_ cylinder with fishing tube; 2 –Adjusted bomb at 350bar; 3 –Extraction cell using 7.5 g of sample (packed) and co-solvent at 50°C (furnace temperature); 4 –Static/dynamics valve; 5 –Restrictor valve adjusted to 6g/min CO_2_ flow; 6 –Extracts into a collection bottle; 7 –Flow meter; 8 –Gas meter; (B) Low pressure extraction (conventional extraction) 1 –Propolis sample in ethanol (80%); 2 –Process extraction in a shaker (70°C, 30 minutes, 710rpm); 3 –Centrifugation at 8800rpm for 11 minutes at 5°C; 4 –Supernatant, centrifugation was repeated with the residue (10 ml of 80% ethanol); 6 –Homogenised supernatants and kept at 50°C until completely dry.

The extraction cell consisted of a packaging using 7.5 g of homogenised propolis sample with 1% ethanol co-solvent (m/m), wool and glass pearls, aimed at avoiding the preferred paths of CO_2_ and the total filling of the bed. The extraction conditions were: pressure of 350 bar, temperature 50°C, S/F of 110 (mass of CO_2[solvent]_ / mass of propolis_[solute]_), 1% co-solvent (ethanol m/m), flow of CO_2_ of 6 g/min and total time of extraction 2 h 30 min [26,43]. The temperature of the restrictor valve was adjusted at 80°C for all extraction processes. The extracts were collected in glass vials of 50 ml, immersed in ice at room pressure. The vials containing the extracts were protected with aluminium foil in inert atmospheric conditions (N_2_) in order to avoid degradation of the material, and kept at 5°C until analysis (Fig 1A).

#### Low pressure extraction (conventional extraction)

The ethanolic extracts of propolis were prepared with the addition of 15 ml ethanol (80%) to 2g propolis. The extraction occurred at 70°C temperature for 30 minutes under constant agitation in a Shaker (MA 420/MARCONI–Brazil) incubator, at 710 rpm rotation. Following that, the extract was centrifuged (Centrifuge SIGMA 2–16 KL) at 8800 rpm for 11 min at 5°C. At the end of the centrifugation, the supernatant was transferred to a 50 ml beaker, and 10 ml ethanol (80%) was added to the tube residue, where centrifugation was repeated. The supernatants were homogenised and kept at 50°C until completely dry (Fig 1B). Afterwards, the extracts were stored in tubes covered in aluminium foil in inert atmospheric conditions (N_2_) in order to avoid degradation of the material. The material was kept at 5°C until analysis [6].

### Chromatographic analysis: identification and quantification of the 3,5-diprenil-4-hidroxicinamic (Artepillin C) and acid 4-hidroxicinamic (p-coumaric acid)

To the identification and quantification of the 3,5-diprenil-4-hidroxicinamic (Artepillin C) and acid 4-hidroxicinamic (p-coumaric acid) in the propolis extracts, firstly, solutions of 10 mg/ml of propolis extracts obtained in the different conditions of the process were prepared and dissolved in ethanol and placed in ultrasound bath (Sanders, SONICLEAN 6 –Minas Gerais, Brazil—ANVISA 80273140001) for 30 minutes (Electronic timer microprocessor–Temperature 35°C electronically controlled and Ultrasound frequency 40 kHz). The exposure to the ultrasound system only started after reaching 35°C. The samples were filtered in a cellulose ester membrane filter 0.45 μm (Micropore®) for posterior injection in the High Performance Liquid Chromatograph (HPLC).

The chromatographic experiments were performed with the system HPLC EZChrom Elite, consisting of a VRW HITACHI L-2130 pump, equipped with an automatic injector and diode arrangement detector (DAD) VRW HITACHI L-2455 and oven VRW HITACHI L-2300. The chromatographic separation was based in the method proposed by Daugsch [15], adapted. The column LiChroCART Purospher StaR® RP18-e (75 mm x 4 mm i.d.) (3 μm) (Merck, Darmastad, Germany) was used together with a pre-column LiChroCART 4–4 LiChrospher 100RP18 (5 μm) from Merck.

The conditions for analysis were performed with an elution gradient with a mobile phase of aseptic acid 5% (aqueous phase) and methanol (organic phase) in different proportions and with total analysis time of 70 minutes (0 min-80:20; 10 min 70:30; 15 min-60:40; 30 min-50:50; 45 min-40:60; 60 min-30:70; 65 min-0:100; 70 min-80:20). The volume of injection was of 10 μL. The equipment was operated at room temperature (25±2°C). The reading of the diode arrangement detector was in the range of 200 to 400 nm and the chromatographic acquisition was defined at 290 nm. The identification of the compounds was performed through the comparison of time of retention and ultraviolet spectrum between the samples and the controls (standard). Aiming to ensure the reliability of the results obtained, a validation took place according to the National Health Surveillance Agency (ANVISA) [44] and National Institute of Metrology, Standardization and Industrial Quality (INMETRO) [45] methodologies. This was done in accordance to the parameters of selectivity, linearity, precision, accuracy, detection limits and quantification limits.

### Determination of the total phenolic compounds

The total phenolic content was determined using the Folin Ciocalteau reagent [46–47]. The reaction was prepared with a 0.5 ml aliquot of propolis extract (dissolved in ethanol aimed at obtaining a concentration of 200 μg/ml), 2.5 ml aqueous solution of Folin-Ciocalteau 10% and 2.0 ml sodium carbonate at 7.5%. The mixture was introduced in a thermo-regulated bath at 50°C for 5 minutes; afterwards, the absorbance was measured in a spectrophotometer LAMBDA 25 UV/vis Systems (PerkinElmer, Washington—USA) at 765 nm. The quantity of total phenolic was expressed as Gallic acid equivalents (EAG) (mg EAG/g of sample) through a calibration curve using known solutions to Gallic acid standard in the same conditions (λ = 765 nm). The Folin Ciocalteu method is associated to the appearance of a blue colouring due to the oxidation of phenols in basic medium [48].

### Determination of flavonoid content

The determination of flavonoid content was performed through the reading in a spectrophotometer (LAMBDA 25 UV/Vis Systems—PerkinElmer USA) at 415 nm, using aluminium chloride at 2% in methanol [49] in a 1:1 solution (extract:aluminium chloride). The same procedure was performed using known solutions of quercetin standard to elaborate a standard curve. The quantity of total flavonoids was expressed as quercetin equivalents (EQ) (mg EQ/g of sample).

### Determination of anti-oxidant activity *in vitro* (2,2-Diphenyl-1-picrylhydrazyl–DPPH)

The anti-oxidant activity *in vitro* of propolis extracts obtained in different conditions was evaluated using the reactive 2,2-Diphenyl-1-picrylhydrazyl (also known as the capacity to sequestrate the radical DPPH) [50–51]. Five dilutions of the extracts were prepared (20 to 400 μg/ml) in triplicates. An aliquot of 1 ml of each extract dilution was transferred to assay tubes with 3.0 ml of the ethanoic solution (Ethanol–absolute alcohol 99.8%) of the radical DPPH (0.004%). After 30 minutes incubation in the dark and at room temperature, the reduction of the free radical DPPH was measured through the reading of absorbance in 517 nm spectrophotometer (LAMBDA 25 UV/Vis Systems–PerkinElmer, Washington—USA).

The same procedure was performed with ethanol replacing the sample, considered blank. The capacity to sequestrate free radicals was expressed as the percentage of oxidation inhibition in the radical and calculated according to Eq 1. The IC_50_ value (necessary concentration of the extract to sequestrate 50% of DPPH radical) was calculated through the line equation based on the concentrations of extracts and its respective percentages of radical DPPH sequestration.

%sequestration=100−[(final absorbance of sample*100)/blank absorbance] (1)

### Determination of antioxidant activity *in vitro*: ABTS method (2,2′-azino-bis-3-ethylbenzthiazoline-6-sulphonic acid)

The ABTS assay was based on the van der Berg et al. [52] method, slightly modified by Kim et al. [53], with adaptations. Initially, a solution at 7 mM was prepared in distilled water. From this solution, an aliquot of 5 ml was removed and 88 μL of potassium persulfate at 2.45 mM was added. The final product was incubated for 16 hours, protected from light in order to enable production of the radical cation ABTS^•+^. Afterwards, the solution was diluted in ethanol until it reached 700±50 absorbance, reading at 734 nm using a spectrophotometer. Originated from the formation of radicals, 20 μL aliquots of stock solutions of 1 mg.ml^-1^, 0.75 mg.ml^-1^, 0.5 mg.ml^-1^ and 0.1 mg.ml^-1^ taken from the extracts were added to 2 ml of the final solution of ABTS^•+^, and after 6 minutes of incubation the samples were read at 734 nm absorbance. The results were expressed in TEAC (antioxidant activity equivalent to Trolox (6-hydroxil-2,5,7,8-tetramethylchromo-2-carboxilic acid)) (Vitamin E).

### Antimicrobial activity of the extracts EtOH and SCO_2_

The antimicrobial activity of the extracts EtOH and SCO_2_ was determined through the Minimal Inhibitory Concentration (MIC) and Minimal Bactericide Concentration (MBC) against *Staphylococcus aureus* (ATCC 33951 and 25923) and *Escherichia coli* (ATCC 25922), according to the *Clinical and Laboratory Standards Institute* [54] and Koo et al. [55]. The strains used were provided by the Bacteria Cultures Collection of the Instituto Oswaldo Cruz—FIOCRUZ (Manguinhos—Rio de Janeiro—Brazil) and initially reactivated in liquid BHI (Brain Heart Infusion) (Sigma-Aldrich Chemical Co.—St. Louis, MO, USA) at 37°C for 24h and then grown in BHI agar plates to inoculum preparation. After bacterial growth, the biomass was removed with the aid of bacteriological loops and suspended in 0.89% NaCl sterile solution, homogenizing the bacterial suspensions until turbidity equivalent to 0.5 McFarland standard scale (equivalent to concentration of 1.5x108 CFU/ml). The volume of 30 μl of the bacterial suspension was inoculated on 30 ml of BHI to give a bacterial concentration of 1-2x105 CFU/ml. In order to determine the MIC, the initial inoculum was 1-2x105 CFU/ml, and the concentrations of the extracts varied from 1600–3.1 μg.ml-1. The MIC was defined as the lowest concentration inhibiting bacterial growth (without visible growth) [55]. As a determining factor of MBC, the surface of the agar BHI (Brain Heart Infusion) was seeded with the samples which did not indicate the presence of visible bacterial growth. MBC was defined as the lowest concentration which did not allow any visible bacterial growth in agar [55]. The assays were realized in triplicate for each concentration of the extracts tested.

### *In vitro* activity of the ethanolic extracts (EtOH) on the strains of tumoral cells B16F10

The cellular strain of murine melanoma, B16F10 (ATCC® CRL-6475™) was kept in a culture medium RPMI 1640 (Gibco®, Life Technologies, Carlsbad, CA, USA), complete with 10% foetal bovine serum (FBS) (Gibco®) and 1% antibiotic solution of penicillin/streptomycin. It was placed in an incubator at 37°C in environment an of 5% CO_2_. The *in vitro* tests of the ethanolic extracts (EtOH) performed on these cells followed the procedure: a solution of 1% Trypsin-EDTA was used to detach the culture of confluent cells from the growth bottle; after 5 minutes the solution of trypsin was inactivated by FBS and after adding the culture medium the material was centrifuged for 10 minutes at 1500 RPM; after discarding the supernatant, the pellet of cells was again suspended in a complete RPMI medium and then the inoculum of cells corresponding to the final concentration of 1x10^5^ cells/ml was calculated. After distributing the culture medium in plaques of 24 wells, the compounds were added to two concentrations of 50 μg/ml and 100 μg/ml, and the DMSO was used as a diluting control and statistic parameter [56]. The cellular proliferation was determined after 24 and 48 hours of incubation using the method of colorimetric assay adapted from Busatti and Gomes [57], and reading by an ELISA (enzyme-linked immunosorbent assay) reader at 570 nm. The cells used in this study were purchased from the American Type Culture Collection (ATCC, Manassas, VA, USA).

### Statistical analysis

The results were expressed in the form of mean ± standard deviation (n = 3). For the statistical analysis of the results, the programme Statistica® 6.0 from StatSoft (Tulsa, USA) was used. Variance analysis (ANOVA) and the Tukey test were used to identify significant differences among the means (p>0.05).

## Results and Discussion

### Characterization of raw propolis samples

Propolis is a complex mixture, containing resins, balsamic products, wax, essential oils, pollen, and microelements, besides other components. The samples presented a characteristic aroma, balsamic and/or resinous, malleable (red) to rigid (brown and green) consistencies at room temperature, with a very heterogeneous granulometry. It is noted that the analysis of the physical-chemical composition is of great importance to determine the quality of the studied material, considering the incorporation of this matrix in food products [58–60]. On Table 2, the results of the physical-chemical characterization of the different samples of propolis are shown. On Table 3 are the results for the content of certain minerals present in the samples.

**Table 2:** Determination of the content of humidity, total solids, total ash, raw protein, total lipids, raw fibre and water activity (aw) of red, green and brown propolis samples collected in different regions of Brazil.

Sample	Humidity (%)	Total solids (%)	Total ash (%)	Protein (%)	Lipids (%)	Aw	Fiber (%)
**RSE**	7.26±0.99^a^	92.74±0.99^a^	1.04±0.11^a^	1.72±0.01^a^	65.74±2.63^a^	0.690±0.01^a^	3.44±0.84^a^
**RAL**	7.03±0.42^a^	92.97±0.42^a^	0.96±0.03^a^	2.30±0.05^b^	66.33±0.01^a^	0.689±0.01^a^	7.66±0.90^b^
**GMG1**	8.84±0.05^b^	91.16±0.05^b^	3.30±0.11^b^	10.58±0.08^c^	45.76±1.77^b^	0.705±0.01^b^	16.36±1.34^c.f^
**GMG2**	9.03±0.48^b.d^	90.97±0.48^b.d^	3.24±0.17^b^	9.83±0.97^c^	47.33±4.82^b.d^	0.704±0.01^b^	15.92±1.03^c^
**GPR**	7.13±0.12^a^	92.87±0.12^a^	3.15±0.03^b^	9.98±0.83^c^	48.72±1.29^b.d^	0.688±0.02^a^	20.89±1.39^d^
**BSC**	7.07±0.10^a^	92.93±0.10^a^	1.73±0.19^c^	3.90±0.49^d^	74.31±5.69^c^	0.657±0.02^c^	7.29±0.30^b^
**BRS**	6.90±0.05^c^	93.10±0.05^c^	0.85±0.03^d^	0.84±0.01^e^	74.08±4.08^c^	0.674±0.01^d^	51.39±0.14^e^
**BPR**	9.16±0.06^d^	90.84±0.06^d^	2.38±0.20^e^	6.90±0.02^f^	49.53±1.70^d^	0.755±0.01^e^	18.11±1.07^f^

Values showing the same letter on the same column do not show significant difference (p>0.05) through the Tukey test at 95% confidence level.

**Table 3:** Quantification of minerals, sodium (Na), potassium (K), lithium (Li) and calcium (Ca) (mg/Kg), from the ash of propolis samples from different regions of Brazil.

Sample	Na (mg/Kg)	K (mg/Kg)	Li (mg/Kg)	Ca (mg/Kg)
**SER**	14.90±0.89^a^	23.70±1.59^a.e^	6.10±0.69^a^	45.10±0.01^a^
**RAL**	10.10±0.41^b^	28.70±3.16^a^	4.50±0.64^b^	40.10±0.72^b^
**GMG1**	2.40±0.01^c^	399.1±4.91^b^	1.80±0.01^c^	9.00±0.01^c^
**GMG2**	2.40±0.01^c^	317.30±13.43^c^	1.80±0.01^c^	8.40±0.01^d^
**GPR**	3.00±0.01^d^	331.70±15.81^c^	1.80±0.01^c^	9.60±0.01^e^
**BSC**	15.30±1.05^a^	110.30±6.77^d^	3.10±1.00^d^	5.90±0.04^f^
**BRS**	6.70±0.70^e^	23.20±0.98^e^	1.90±0.08^e^	7.40±0.08^g^
**BPR**	1.10±0.11^f^	5.70±0.68^f^	3.40±0.01^d^	29.60±0.62^h^

Values showing the same letter on the same column do not show significant difference (p>0.05) through the Tukey test at 95% confidence level.

The value of humidity and total solids varied from 6.90±0.05 (BRS) to 9.16±0.06% (BPR) and from 90.84±0.06 (BPR) to 93.10±0.05% (BRS), respectively, among the samples. Two samples of green propolis (GMG_1_ and GMG_2_) and one from brown propolis (BPR) were out of the required standards for the humidity content (maximum of 8%) [61]. As expected, the higher values of aw were identified on the samples with high humidity. In relation to the contents of ash, protein, lipids and fibres, a significant variation was observed among the samples (p>0.05), with results varying from 0.85±0.03 (BRS) to 3.30±0.11% (GMG_1_), 0.84±0.01 (BRS) to 10.58±0.08 (GMG_1_), 45.76±1.77% (GMG_1_) to 74.31±5.69% (BSC) and 3.44±0.84 (RSE) to 51.39±1.03% (BRS), respectively (Table 2).

The determination of the total ash content is particularly important in samples of propolis commercialized in powder form, as this analysis can identify a possible adulteration of the material through the presence of impurities, or even residues from previously extracted propolis [6]. The samples were within the limit established by the Brazilian legislation (maximum 5%) [61]. Among the microelements analysed and identified in the samples, we can highlight the high contents of potassium on the three samples of green propolis (Table 3). Some studies show aluminium, vanadium, iron, calcium, silicon, manganese, strontium and potassium, as the main microelements present in propolis samples [5,49,62–64].

Performing a comparative evaluation of samples of the same type (in relation to colour), the lower variation identified for the physical-chemical parameters studied was for the red propolis (RSE and RAL). There are a few works reporting the total physical-chemical characterization of propolis, however the values obtained in this study for certain parameters are similar to those found in the literature [24,64–68]. The variation identified among the samples studied and with those from other studies is easily explained by the type of propolis, flora of the region and period of collection.

**Fig 2:**
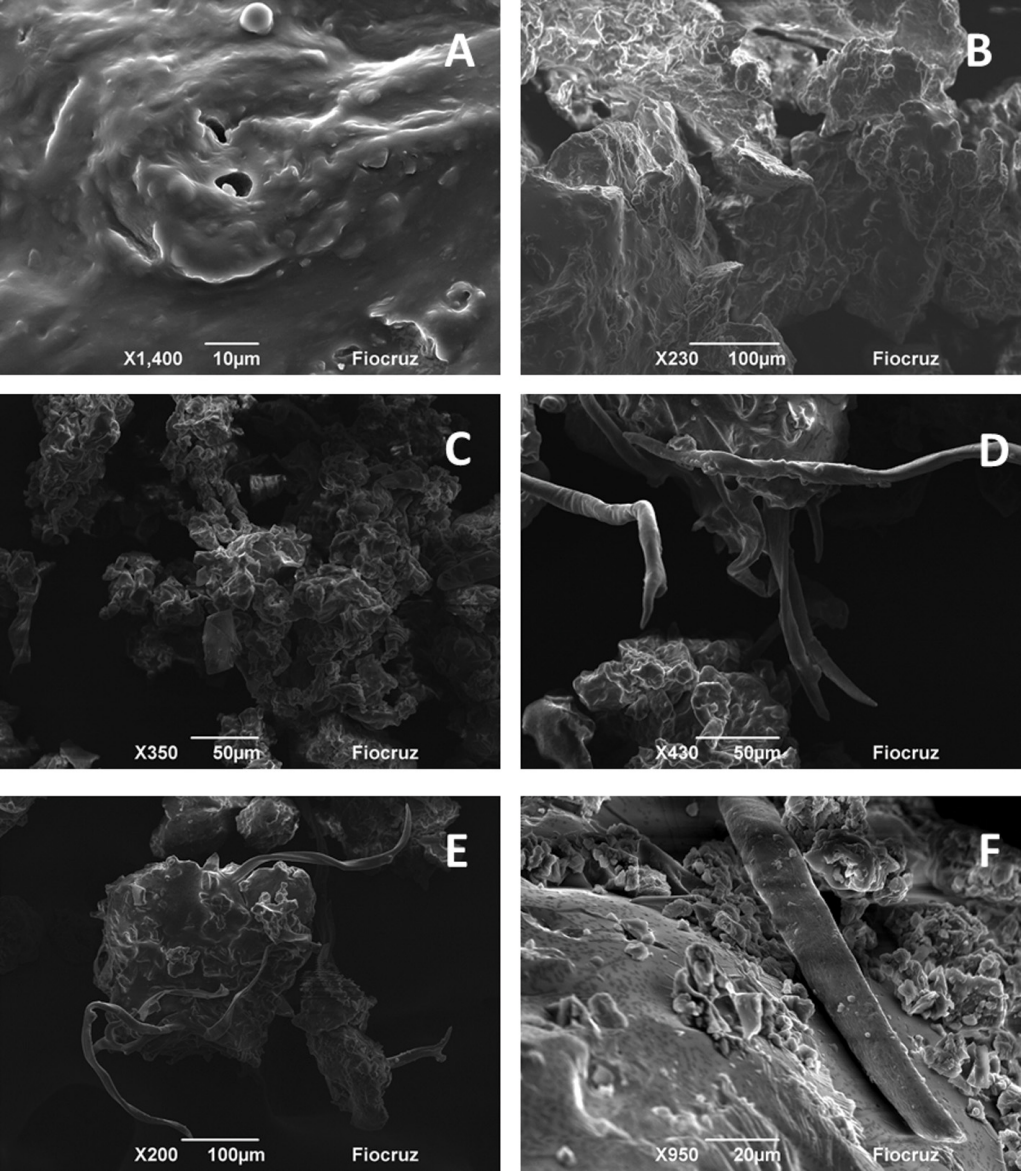
Images obtained by Scanning electron microscopy (SEM) for propolis samples. A–RSE; B–RAL; C–GMG_1_; D–GMG_2_; E–GPR; F–BSC; G–BPR; H–BRS.

On Fig 2 the micrographics obtained for the different propolis samples are shown. No study evaluating the Brazilian propolis by SEM was identified. On the visual and microscopic analysis no strange substances were identified. In all images, it is possible to observe rugged surfaces covered by layers of wax and extractives. Similar characteristics were identified by Tylkowski et al., [69] for samples of propolis from Bulgaria. It is also important to note that there were similarities identified on the microscopic appearance (profile) of the samples of the same type (colour). For example, in all samples of green propolis, vegetable constituents were found, probably tector and/or glandular trichome and resinous substances from the vegetative apices of *Baccharis dracunculifolia* (Fig 2C, 2D and 2E) [7,70–72]. Elements which are similar to vegetable parts were also identified on the samples of brown propolis, which probably come from the flora visited by bees, such as species of *Copaifera* (Fig 2F, 2G and 2H) [73].

### Determination of content for phenolic compounds, flavonoids and antioxidant activity of EtOH and SCO_2_ activity

**Fig 3:**
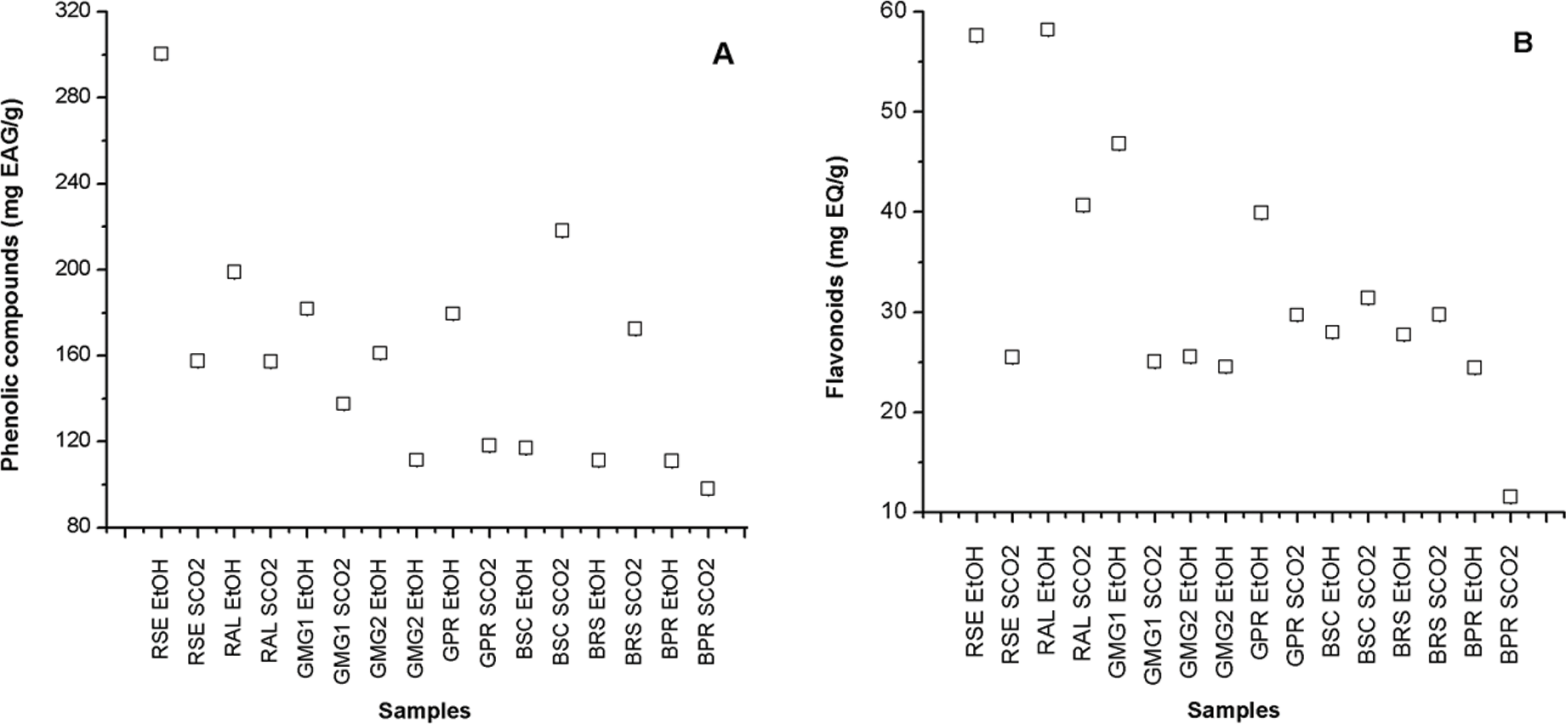
Content of total phenolic compounds expressed in mg EAG/g (A) and of flavonoids expressed in mg EQ/G (B) of the extracts of different samples of Brazilian propolis. SCO_2_ –Extracts obtained by SFE; EtOH–Extracts obtained by ethanolic extraction; Lower values of IC_50_ indicate a higher activity of radical elimination; Average of analysis obtained in triplicate (n = 3).

**Fig 4:**
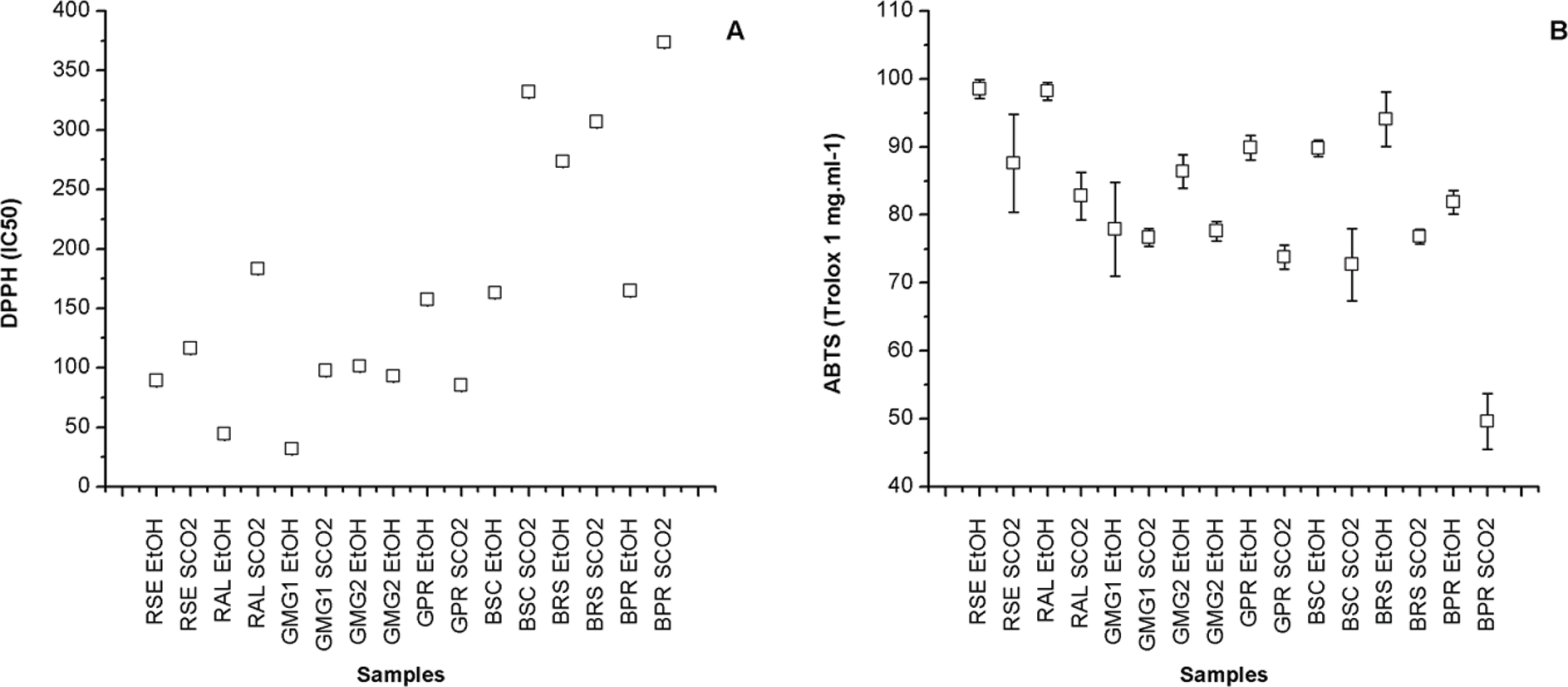
Determination of antioxidant activity of the extracts from different samples of Brazilian propolis, by DPPH (IC_50_) (A) and ABTS (Trolox 1 mg.ml^-1^) (B). SCO_2_ –Extracts obtained by SFE; EtOH–Extracts obtained by ethanolic extraction; Average of analysis obtained in triplicate (n = 3).

Table 4 shows the results for the content of total phenolic compounds, flavonoids and antioxidant activity of the extracts from different samples of propolis obtained by the two extraction methods (conventional ethanolic–EtOH and supercritical–SCO_2_). The content of phenolic compounds varied from 97.97±0.01 (BPR SCO_2_) to 300.36±0.01 mg EAG/g (RSE EtOH), whereas the content of flavonoids varied from 11.55±0.01 (BPR SCO_2_) to 58.19±0.01 mg EQ/g (RAL EtOH) among other samples (Fig 3). There is great controversy in relation to the content of flavonoids present in samples of Brazilian propolis, in which phenolic acids are generally a lot more abundant. The antioxidant activity varied from 373.53±0.15 (BPR SCO_2_) to 31.80±0.16 (GMG_1_ EtOH) for DPPH (IC_50_) and from 49.60±4.10 (BPR SCO_2_) to 98.50±1.40 (RSE EtOH) for ABTS (Trolox 1 mg.ml^-1^) (Fig 4).

**Table 4:** Determination of the content of total phenolic compounds (mg EAG/g), flavonoids (mg EQ/g), antioxidant activity by DPPH (IC_50_) and ABTS (%) of the extracts of different samples from Brazilian propolis obtained by ethanolic extraction (EtOH) and by SFE (SCO_2_).

Samples	Phenolic Compounds(mg EAG/g)	Flavonoids(mg EQ/g)	DPPH(IC_50_)	ABTS (%)(Trolox 1 mg.ml^-1^)
**RSE EtOH**	300.36±0.01^a^	57.60±0.01^a^	89.32±0.28^a^	98.50±1.40^a^
**RSE SCO**_**2**_	157.43±0.01^b^	25.46±0.01^b^	116.49±0.23^b^	87.60±7.20^b.d^
**RAL EtOH**	198.77±0.01^c^	58.19±0.01^c^	44.29±0.29^c^	98.20±1.30^a^
**RAL SCO**_**2**_	157.16±0.01^b^	40.65±0.01^d^	183.11±0.31^d^	82.80±3.50^b^
**GMG**_**1**_ **EtOH**	181.71±0.01^d^	46.80±0.01^e^	31.80±0.16^e^	77.90±6.80^c^
**GMG**_**1**_ **SCO**_**2**_	137.52±0.01^e^	25.02±0.01^b^	97.74±0.22^f^	76.70±1.29^c^
**GMG**_**2**_ **EtOH**	160.98±0.01^f^	25.52±0.01^b^	101.45±0.23^g^	86.40±2.48^b^
**GMG**_**2**_ **SCO**_**2**_	111.33±0.01^g^	24.52±0.01^f^	93.02±0.20^h^	77.60±1.39^c^
**GPR EtOH**	179.52±0.01^h^	39.90±0.01^g^	157.39±0.26^i^	89.90±1.80^d^
**GPR SCO**_**2**_	118.14±0.03^i^	29.71±0.01^h^	85.34±0.23^j^	73.80±1.80^e^
**BSC EtOH**	117.03±0.01^j^	27.97±0.01^i^	163.00±0.31^l^	89.80±1.20^b.d^
**BSC SCO**_**2**_	218.09±0.01^l^	31.38±0.01^j^	331.88±0.09^m^	72.70±5.30^f^
**BRS EtOH**	111.25±0.01^g^	27.72±0.01^i^	273.46±0.24^n^	94.10±4.00^g^
**BRS SCO**_**2**_	172.43±0.01^m^	29.72±0.01^h.l^	306.91±0.09^o^	76.80±1.10^c^
**BPR EtOH**	110.92±0.01^n^	24.40±0.01^f^	164.52±0.34^p^	81.90±1.73^b^
**BPR SCO**_**2**_	97.97±0.01^o^	11.55±0.01^m^	373.53±0.15^q^	49.60±4.10^h^

Values showing the same letter on the same column do not show significant difference (p>0.05) through the Tukey test at 95% confidence level. EtOH–Extracts obtained by ethanolic extraction; SCO_2_ –Extracts obtained by SFE (CO_2_ as supercritical fluid); Lower values of IC_50_ indicate higher activity of radical elimination.

It was verified that the extracts obtained from red propolis (RSE and RAL) originated from the Brazilian northeast showed the highest content of phenolic compounds and flavonoids. This confirms the biological potential of this “new” type of propolis [4,74–76]. The highest quantity of total phenols, flavonoids and the best antioxidant activity by ABTS was identified in the extract of red propolis from the state of Sergipe (SER EtOH), with values of 300.36±0.01 mg EAG/g, 57.60±0.01 mg EQ/g and 98.50±1.40%, respectively (Table 4). However, the best results for the antioxidant activity by DPPH was shown on the extract of green propolis from the state of Minas Gerais–GMG_1_ EtOH (IC_50_ of 31.80±0.16).

These results indicate that the total concentration of phenolic compounds or flavonoids is not the only factor responsible for antioxidant properties. The chemical nature of the phenolic compounds and, perhaps, the presence of other compounds contribute to the total antioxidant capacity of the extracts [77]. The extracts EtOH and SCO_2_ obtained from brown propolis showed the lowest values of phenols, flavonoids and antioxidant activity, therefore showing the lowest biological potential of this type of propolis when compared to the samples of green and/or red Brazilian propolis evaluated in the study.

Frozza et al. [32] identified a content of 151.55±1.95 mg/g of phenolic compounds and a IC_50_ of 270.13±24.77, whereas Alencar et al. [18] found a content of 232.00±22.30 mg/g for total phenols, 43.00±1.00 for flavonoids and a IC_50_ of 57.00±3.20, for ethanolic extracts of red propolis from Sergipe and Alagoas (Brazil), respectively. Cottica et al. [77] determined values of DPPH (IC_50_) among 47 and 160 ng/ml in extracts of hydro alcohols from the Brazilian green propolis, whereas Christov et al. [78] found values between 65 and 79% of inhibition by DPPH for ethanolic extract of Canadian propolis at 210 ng/ml. The results identified in this study are in accordance with the literature [79].

Significant differences were identified (p>0.05) for the results of the compounds analysed (Table 4), when compared to the extraction method for the same sample, as well as for the extracts obtained through the same method and samples of different types. The variations identified among the samples (p>0.05) were already expected, considering that the propolis obtained from different phytogeography regions exhibit very distinct chemical profiles [80–81]. The results found in this study confirm the influence of the origin of the raw material on the composition and characteristics of the extracts.

Chaillou and Nazareno [82] also observed significant (p>0.05) differences on the content of phenolic compounds, flavonoids and antioxidant activity, when evaluating different samples of propolis originated from Santiago del Estero, Argentina, where the averages varied from 92 to 187 mg/g for polyphenols, from 6 to 18 mg/g for flavonoids, and from 49.5 to 65.7% for DPPH. Similar results were also identified by Kumazawa et al. [83], by Kalogeropoulos et al. [84] and Choi et al. [85], when propolis from different geographic origins were evaluated. It stands out that the variability found between the content of compounds and antioxidant activities of propolis from Brazil is attributed to the differences observed in the arboreal species for each geographic area, being justified by the great Brazilian diversity.

Comparatively evaluating the results obtained for the extracts EtOH and SCO_2_ of the same sample, it is noted that the majority of extracts EtOH present the best results for the content of total phenolic compounds and flavonoids, with the exception of the samples BSC and BRS, where the extracts SCO_2_ shows superior values of these compounds. Generally, the extracts obtained by ethanolic extraction (EtOH) show the best antioxidant activities through the methods DPPH and ABTS.

Similar results were observed by Miguel et al. [86] and Cottica et al. [77], who found higher values of total phenols and flavonoids in EtOH extracts of Portuguese and Canadian propolis, respectively, in relation to the aqueous extracts. Zordi et al. [87] also identified higher concentrations in EtOH extracts of Italian propolis when compared to the extracts obtained by SFE in different conditions of process and using CO_2_. Lee et al. [1] also verified that the extracts of Brazilian propolis obtained by SFE showed a lower antioxidant capacity (DPPH) when compared to the extracts obtained by Soxhlet, hot extraction and by ultrasound. However, Laskar et al. [88] reported that the phenolic compounds in aqueous extracts of Indian propolis were in higher concentration when compared to EtOH extracts.

The lower concentrations of phenolic compounds, flavonoids and antioxidant activity identified in the extracts obtained by SFE (SCO_2_) confirms the higher presence of undesirable substances such as wax, resin and other materials present in propolis, which could indicate a lower biological potential for these extracts [24]. Wax and other organic detritus are removed during the process of ethanolic extraction, and the propolis extracts obtained that way can contain the majority of the antioxidant constituents [84].

Zordi et al., [87] indicated two possible applications for the use of supercritical CO_2_ for samples of propolis: obtain lipophilic fractions enriched by specific components, or as a pre-treatment of the raw material to facilitate the additional extraction with ethanol. As described by Biscaia and Ferreira [24], complex natural matrices such as propolis, can result in different products, depending on the method used. Therefore, the viability of the process is related to the yield and quality of the product, in order to improve the biological potential present in the raw material. With that, the efficacy of the extraction method (higher or lower selectivity) for obtaining total phenolic compounds and flavonoids can vary according to the origin and composition of the raw material.

### Quantification of Artepillin C and p-coumaric acid in EtOH and SCO_2_ extracts

**Fig 5:**
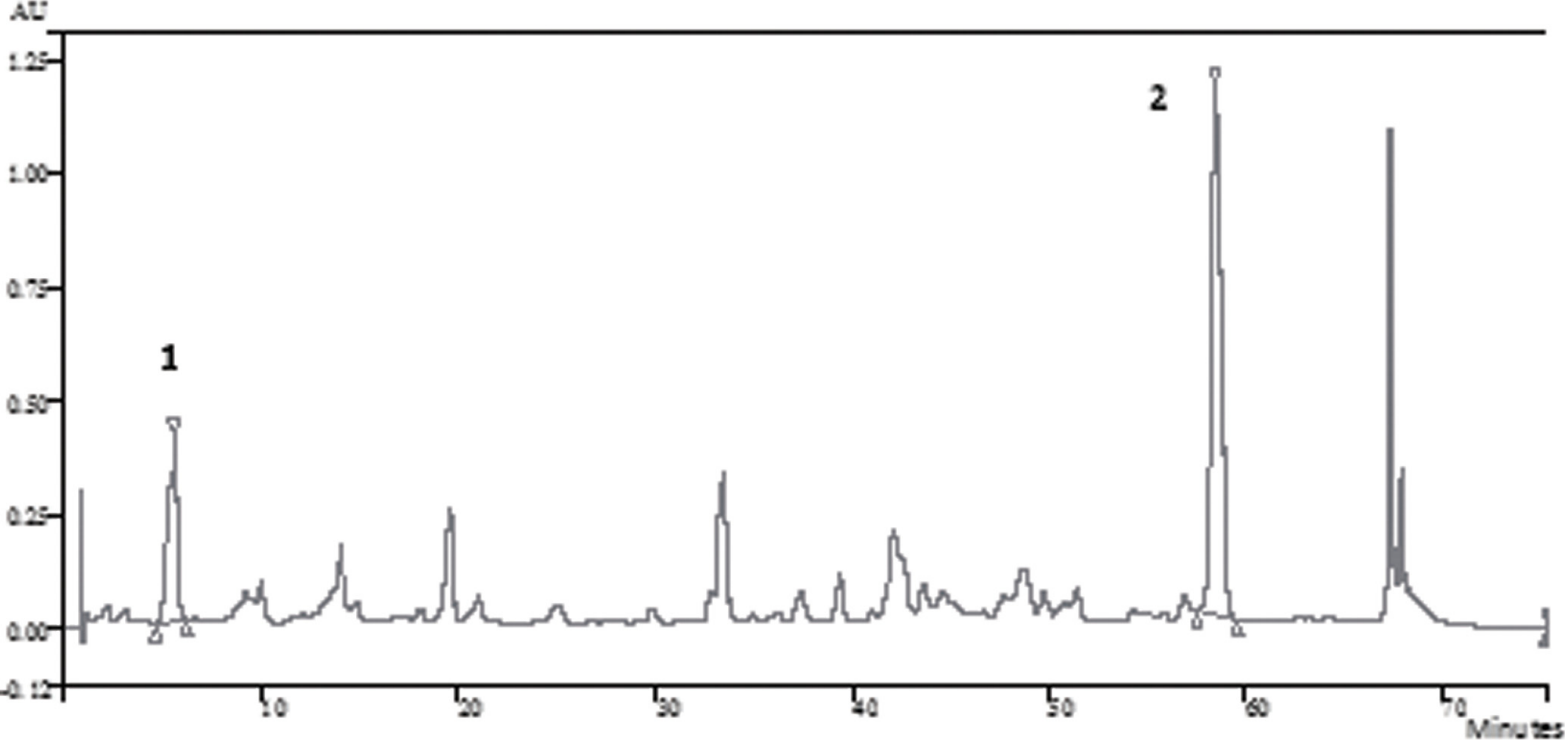
Chromatograms of green propolis ethanolic extract from Paraná (GPR EtOH)–(1) p-coumaric acid; (2) Artepillin C.

The results of the quantitative analysis of Artepillin C and p-coumaric acid on the extracts of the different samples of Brazilian propolis are shown on Table 5. As expected, the markers Artepillin C and p-coumaric acid were present in all extracts of green propolis [1,7,10,89–91]. The Fig 5 shows the chromatogram of a green propolis samples obtained by ethanolic extraction (GPR EtOH). Artepillin C was also identified in two samples of brown propolis originated from the regions of Santa Catarina (SC) and Paraná (PR). As described by Lee et all. [1], different types of propolis can contain a varied quantity of Artepillin C, however the green propolis, originated from the vegetal species *Baccharis dracunculifolia* shows a higher quantity of this compound.

**Table 5:** Determination of the content of 4-hidroxicinamic acid (p-coumaric acid) and 3,5-diprenil-4-hidroxicinamic (Artepillin C) of extracts from different samples of Brazilian samples obtained by ethanolic extraction (EtOH) and by SFE (SCO_2_).

Samples	p-coumaric acid (μg/ml)	Artepillin C (μg/ml)
RSE EtOH	----	----
RSE SCO_2_	----	----
RAL EtOH	----	----
RAL SCO_2_	----	----
GMG1 EtOH	24.65±0.24^a^	569.85±0.11^a^
GMG1 SCO_2_	195.12±6.12^b^	798.05±1.20^b^
GMG2 EtOH	26.64±1.56^a^	340.89±1.11^c^
GMG2 SCO_2_	101.68±2.87^c^	539.22±2.23^d^
GPR EtOH	35.57±3.45^d^	464.49±9.23^e^
GPR SCO_2_	198.09±3.12^e^	845.05±0.12^f^
BSC EtOH	----	58.32±1.00^g^
BSC SCO_2_	5.05±0.10^f^	106.81±1.08^h^
BRS EtOH	----	----
BRS SCO_2_	----	----
BPR EtOH	----	82.67±6.12^i^
BPR SCO_2_	----	315.96±5.89^j^

Values showing the same letter, in the same column, do not show significant differences (p>0.05) by the Tukey test at 95% confidence interval; EtOH–Extracts obtained by ethanolic extraction; SCO_2_ –Extracts obtained by SFE (CO_2_ as supercritical fluid); -—Not identified.

The content of p-coumaric acid varied from 5.05±0.10 (BSC SCO_2_) to 198.09±3.12 μg/ml (GPR SCO_2_), whereas that of the Artepillin C varied from 58.32±1.00 (BSC EtOH) to 845.05±0.12 μg/ml (GPR EtOH) among the extracts. The results show differences among the samples (p>0.05), which are in conformity with its place of origin. The green propolis from Paraná (PR) was the one which presented the highest values of the studied compounds. Kumazawa et al. [83] identified the presence of p-coumaric acid and Artepillin C (43.9 mg/g) in propolis from Brazil when evaluating samples from different countries.

It was also identified that, from the 16 samples analysed, the Artepillin C was only present on the green propolis from Brazil. Tazawa et al. [92] also concluded that the p-coumaric acid and Artepillin C are the main active components of the Brazilian propolis, whereas certain flavonoids are the main constituents of propolis from other countries (China, Japan and Bulgaria, among others). Shimizu et al. [93] identified a high quantity of Artepillin C (21.0 mmol/100g) and p-coumaric acid (7.70 mmol/100g) in the Brazilian propolis from Minas Gerais. Ahn et al. [94] evaluated samples of propolis collected from different regions of China and observed the presence of p-coumaric acid in all samples, which varied from 2.3 to 42.3 mg/g.

It becomes important to note that the extraction with supercritical CO_2_ (SCO_2_) was significantly more efficient for obtaining both analysed markers, when compared to the same sample (p>0.05). The extracts obtained by SFE can have a concentration four times higher than that of the p-coumaric acid, when compared to the EtOH extracts. Generally, despite this not being the most efficient method for obtaining the total phenolic compounds and antioxidant activity of the samples, it is clear that it provides a higher selectivity to obtain both analysed compounds, Artepillin C and p-coumaric acid in samples of Brazilian green propolis.

It is important to highlight that one of the main aspects that should be considered in SFE is the choice of operational conditions in the process of extraction, because that it can provide an additional advantage to the conventional methods, considering the lowest total yield and the highest cost. Besides that, the use of the optimized values for the different conditions can significantly improve the yield and the recovery of the target compound [30].

In this work, the optimized conditions of temperature, pressure, percentage of co-solvent and quantity of sample of a previous work from our group [43], which specifically evaluated the obtainment of Artepillin C and p-coumaric acid. In view of that, a better extraction of both phenolic acids by SFE is justified, when compared to EtOH extraction.

Similar results were identified by other authors, when the extraction of relevant compounds from different natural matrices using SFE and conventional extraction were compared. Those studies have shown the selectivity of SFE, generating products with higher biological value [31,95–99].

For example, Lee et al. [1] individually investigated organic solvents (conventional methods) and supercritical CO_2_ to recover Artepillin C from the Brazilian propolis, and identified that the extracts obtained by SFE (45.3±0.10 mg/ml) showed the highest contents of the relevant compound (Soxhlet = 16.9±0.2 0mg/ml; Hot extraction = 16.4±0.23 mg/ml; Ultrasound = 16.0±0.06 mg/ml). Sun et al. [100] extracted the active substance paeonol from *Cynanchum paniculatum* by SFE and other conventional techniques, identifying 72.02% of the active substance in the extracts obtained by SFE, a highly superior result to that found by other methods of extraction (ultrasound 1.56%, steam distillation 1.64% and Soxhlet 2.74%).

The patent CN 1258511 (Chinese) and BR 1020140320121 (Brazilian) describe the extraction of active compounds from propolis by SFE. There, it is shown that the extracts obtained using CO_2_ as supercritical fluid (and ethanol as co-solvent) are rich in different compounds (phenolic acids, flavones and terpenes) [26,101], being, therefore an efficient method for yield and selectivity for the extractive process of relevant compounds from propolis. Extracts of propolis obtained with supercritical fluids are already being sold in the markets of Japan, considering the proof of anti-tumour properties of these extracts [102].

### Determination of the antimicrobial activity of the extracts EtOH and SCO_2_

Table 6 shows the values of MIC and MBC obtained for the different SCO_2_ and EtOH extracts of the propolis samples tested. It was noted that all extracts showed activity against gram-positive bacteria *Staphylococcus aureus* (ATCC 33951 and 25923) and gram-negative *Escherichia coli* (ATCC 25922), but this effect was dependent on the origin of the matrix and method of extraction. The control sample did not affect the growth of tested bacteria (data not shown). As expected, the extracts from different samples of propolis showed a higher activity against the gram-positive strains than against the gram-negative strains. These results are in accordance with those from Koru et al. [103], Vardar-Ünlü et al. [104], Kim and Chung [105] and Silva et al. [106], which can easily be explained by the structural differences of the bacterial cellular wall [107–108].

**Table 6:** Determination of Minimal Inhibitory Concentration (MIC) and Minimal Bactericide Concentration (MBC) of the extracts from different samples of Brazilian propolis obtained by ethanolic extraction (EtOH) and by SFE (SCO_2_).

	*Staphylococcus aureusATCC 25923*		*Staphylococcus aureus ATCC 33591*		*Escherichia coli* ATCC 25922	

Samples	CIM (μg/mL)	CBM (μg/mL)	CIM (μg/mL)	CBM (μg/mL)	CIM (μg/mL)	CBM (μg/mL)
**RSE EtOH**	50–25	800–400	400–100	1600–800	400	1600–800
**RSE SCO**_**2**_	100–50	800	600–200	1600	800	1600
**RAL EtOH**	100–50	800–400	400–100	1600–800	400	1600
**RAL SCO**_**2**_	200–400	1600–800	800–400	1600	800	1600
**GMG**_**1**_ **EtOH**	200	800	1600–50	>1600	1600–400	>1600
**GMG**_**1**_ **SCO**_**2**_	400	1600	1600–400	>1600	1600–800	>1600
**GMG**_**2**_ **EtOH**	400–200	1600–800	800–200	1600	1600–800	>1600
**GMG**_**2**_ **SCO**_**2**_	800–400	1600	800–400	>1600	1600	>1600
**GPR EtOH**	400–200	1600–800	800–200	1600	1600–800	>1600
**GPR SCO**_**2**_	800–400	1600	800	>1600	1600	>1600
**BSC EtOH**	800–400	1600–800	>1600	>1600	1600–800	>1600
**BSC SCO**_**2**_	800	1600	>1600	>1600	1600	>1600
**BRS EtOH**	800–400	>1600	>1600	>1600	1600–800	>1600
**BRS SCO**_**2**_	1600–800	>1600	>1600	>1600	1600	>1600
**BPR EtOH**	400–200	1600–800	800–200	1600	1600–800	>1600
**BPR SCO**_**2**_	800–400	1600	1600–800	>1600	1600	>1600

When compared to the extraction method, the EtOH extracts showed the best antimicrobial activities, and as previously shown, these extracts also had the best antioxidant activities and the highest content of total phenolic acids and flavonoids. Propolis samples from different regions of Europe and the Middle East were evaluated in a study performed by Popova et al. [109] and a negative correlation between the concentration of phenolics in the extract and MIC was identified. The test was performed with hydro alcoholic extracts of propolis against *Staphylococcus aureus*, and it was found that the higher concentration of phenolics, the more powerful the activity against this bacteria. Jug et al. [110] also evaluated the antibacterial and antifungal efficiency of propolis extracts obtained by different extraction techniques and identified that the EtOH extract showed the best antimicrobial potential.

The extract that showed the highest antimicrobial activity *in vitro* for the three tested strains was the RSE EtOH, which also showed the highest content of phenolic compounds and high values of flavonoids. Among the samples evaluated, the extracts obtained from the samples of red propolis showed the best antimicrobial activities. Koo et al. [55] evaluated extracts of propolis from different types and regions of Brazil (red propolis from Bahia and green propolis from Minas Gerais and Paraná), identified differences in the MIC and MBC for each extract in relation to *Streptococcus mutans*, *S*. *sobrinus* and *S*. *cricetus*, and also that the best results were shown by the red propolis from the Brazilian northeast, as identified in this study.

Alencar et al. [18] also identified a noticeable antimicrobial activity for ethanolic and chloroformic extracts of Brazilian red propolis (Alagoas) against the *Staphylococcus aureus* ATCC 25923 (MIC of 50–100 and MBC of 200–400 –EtOH extract; MIC of 200–400 and MBC of 100–200 –chloroformic extracts) and *Staphylococcus mutans* UA159. It were concluded that the best antimicrobial activity was found for the extract with the highest concentration of chloroformic total phenols. For the extracts from green propolis, the samples GMG_1_ and GPR showed the best antimicrobial potential. The extracts of brown propolis, which showed the lowest antioxidant potentials, presented the highest concentrations for the inhibition of antimicrobial growth for the strains tested. As expected and identified in other studies [18,109,111], the MBC for all extracts was four times superior to the MIC.

As already described in other studies, and also identified in this work, regardless of its geographic origin, propolis shows an important antimicrobial activity, since this property is essential for the preservation and maintenance of the hive [84,103,112–114]. Different studies show that the antimicrobial activity of this matrix is mainly due to complex synergic effects between the flavonoids, phenolic acids and its derivatives, which are mainly present in propolis [39,115–116].

For example, when the propolis extracts originated from Brazil and Bulgaria were evaluated against strains of *Staphylococcus aureus*, it was found that the Brazilian extracts showed the best antimicrobial potential, which had the highest concentration of phenolic compounds [117]. Although the mechanism of action for the antimicrobial effect of propolis is still not clearly understood and defined, some studies suggest that certain constituents can interfere in the process of bacterial cell division through disorganizing the cytoplasm, causing cellular lysis [118–119]. It was also found in this study, as previously reported by other authors, that the antimicrobial activity of propolis extracts is related to the method of extraction and type of solvent used [110,120]. Therefore, the determination of MIC and MBC is extremely important to evaluate the quality of the extracts and propolis-based products [103,105,108], considering the great variability in its composition.

### Determination of antitumoral activity *in vitro* of EtOH extracts

The present study also investigated the antitumoral activity of the EtOH extracts of the eight samples of propolis against the cellular strains of melanoma murine (B16F10), evaluating the anti-proliferative effects. Generally, the ethanol extracts showed the best results for content of phenolic compounds, flavonoids and antioxidant activity (DPPH and ABTS). Because of that, those extracts were selected for analysis *in vitro* against human cancer cell lines B16F10. Moreover, the cost associated with these tests do not enables the evaluation of extracts obtained by supercritical fluid extraction. Therefore, comparative and statistical analysis was performed only between different samples of propolis, considering only an ethanol extraction method.

**Fig 6:**
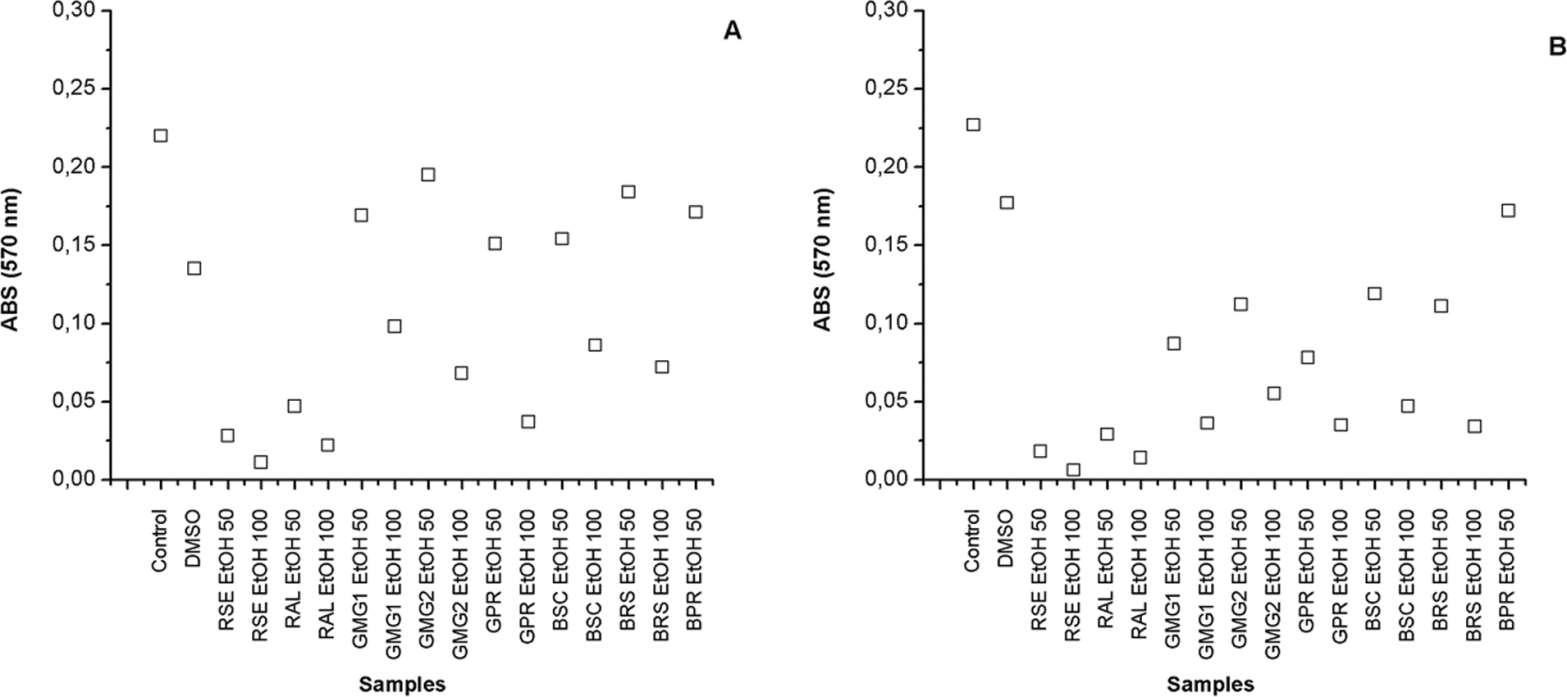
Activity of the EtOH extracts of different samples of Brazilian propolis on the cellular proliferation of the strain B16F10 (murine) after 24 (A) and 48 (B) hours of incubation on both concentrations tested (50 and 100 μg/ml).

On Fig 6A and 6B are shown the activity on the cellular proliferation of the strain B16F10 after 24 and 48 hours of incubation on both concentrations tested (50 and 100 μg/ml). After 24 and 48 hours of incubation, all extracts showed a significant inhibition of cellular proliferation in comparison to the control (p>0.05).

The best results were shown by the extracts derived from the red propolis from the northeast region of Brazil, that is, Sergipe (RSE EtOH) and Alagoas (RAL EtOH). After 48 hours of incubation, the extracts RSE EtOH with concentrations of 50 and 100 μg/ml showed the lowest contents of viable cells, showing absorbance values of 0.018±0.002 and 0.006±0.001 (1x10^5^ cells/ml), respectively. Among the green propolis extracts studied, the sample originated from Paraná, in Brazil (GPR EtOH) showed the best results for proliferative inhibition of cells B16F10. This extract also showed the highest concentration of Artepillin C and p-coumaric acid (Table 6).

The extracts showing the lower potential were those obtained from the brown propolis. The results found for antitumoral activity are in accordance to those previously identified for the content of antioxidant compounds (Table 5). Similar results to this study were found by Franchi-Jr et al. [121], when identifying that the *in vitro* cytotoxic activity of ethanolic extracts of red propolis against strains of human leukemic cells was superior when compared to the extracts of green propolis. Popolo et al. [122] also identified anti-proliferative effects of the ethanolic extract of the brown propolis from Cuba in cellular lineages of human breast cancer.

The best anti-proliferative effect showed by the red propolis extract, when compared to other samples of propolis may depend on its differentiated composition, for example, the presence of formononetin, the main isoflavones found in this type of propolis [123–124]. Recently, a polyisoprenylated benzophenone (xanthochymol) was also identified in the red propolis [123]. Different studies point to xanthochymol and formononetin as showing activity against tumoral cells [125–128].

It is likely that the anti-proliferative activity of this type of propolis occurs through the mechanism of cellular circle halt and apoptosis, as indicated in some studies [121,129–130]. Novak et al. [56] identified that an active fraction of the ethanolic extract of a sample of the Brazilian red propolis (João Pessoa) containing xanthochymol and formononetin showed superior anti-proliferative effects in strains of B16F10 cells, when compared only with the ethanolic extract of the sample.

In previous studies with the red propolis of the same geographic origin (Sergipe–RSE) different antitumoral effects against strains of bladder cancer cells and the presence of formononetin in its composition have been reported [76], and on larynx, uterus and kidney cancer cells [32]. López et al. [125] evaluated samples of red propolis from different regions and identified the presence of formononetin in every sample analysed, among which, four samples were from the state of Sergipe and two from Alagoas (Brazil).

Despite the fact that the green propolis, especially the GPR EtOH, show a lower potential when compared to the extract of red propolis (RSE and RAL), the inhibition in the presence of B16F10 cells was also very significant and relevant (Fig 5), especially when compared to the control (p>0.05). The presence of Artepillin C, a substance found in the Brazilian propolis, is the main constituent of the extracts of green propolis, followed by p-coumaric acid [83,94,131] and also attested by this work.

Carvalho et al. [132] also identified the anti-tumoral effects *in vitro* of the ethanolic and oily extracts of the green propolis from Paraná (Brazil), against different strains of human tumoral cells: HL-60 (leukaemia), HCT-8 (colon), MDA/MB-435 (breast) and SF-295 (brain). Kimoto et al. [133] identified that Artepillin C showed powerful cytocidal effects and induced levels of apoptosis in all the cellular lines of human leukaemia of different phenotypes evaluated. Kimoto et al. [89] identified that Artepillin C from Brazilian propolis showed cytotoxic effects and inhibited the growth of malign murine tumoral cells (B16F10) *in vitro* and *in vivo*, and that the mechanism of action is through the activation of the immunological system.

Matsuno et al. [134] reported that the Artepillin C is one of the most effective anti-tumoral compounds in green propolis, and Kimoto et al. [135] showed that this compound is capable of reducing the tumoral load in certain animal models. Akao et al [136] identified that the anti-tumoral inhibitory effects of the p-coumaric acid were less powerful than those of Artepillin C, and that the compounds available induced apoptosis in the cells characterized by nucleosome and DNA fragmentation analysis. Due to these biological properties, the propolis containing Artepillin C is considered a high quality propolis and the concentration of this component is already been used for the quality control in certain companies [67,132]. From the results identified, and together with other studies, the Brazilian propolis, especially the red and green, can be considered as an important source of active compounds for the development of new drugs with anti-tumoral potential.

## Conclusions

In this study, it was determined that the total quantity of phenolic compounds, flavonoids and antioxidant activity are important parameters to evaluate the quality and biological potential of extracts from the Brazilian propolis, especially considering the great Brazilian biodiversity. The results identified significant differences among the samples (p>0.05), which are in conformity with their place of origin. Despite this chemical diversity, all the types of propolis showed a significant antimicrobial activity, and in most cases, it can be assumed that the compounds responsible are the phenolic constituents of propolis. In relation to the extraction method, generally, the ethanolic extraction (EtOH) was the most efficient for obtaining extracts with the highest content of antioxidant compounds and biological activity. However, the extraction with supercritical CO_2_ (SCO_2_) was the most efficient for obtaining Artepillin C and p-coumaric acid, evidencing the higher selectivity of SFE for obtaining both important markers for the Brazilian green propolis.

Finally, it must be noted that, due to its valuable properties and high biological potential, also already evidenced in other studies, the propolis can be considered as an important source of natural antioxidant compounds. New studies about the propolis complete chemical composition are under way.

## References

[1] Lee YN, Chen CR, Yang HL, Lin CC, Chang CMJ. Isolation and purification of 3,5-diprenyl-4-hydroxycinnamic acid (artepillin C) in Brazilian propolis by supercritical fluid extractions. Sep Purif Technol. 2007; 54: 130–138. 10.1016/j.seppur.2006.08.028

[2] Toreti VC, Sato HH, Pastore GM, Park YK. Recent progress of propolis for its biological and chemical compositions and its botanical origin. Evid Based Complement Alternat Med. 2013; 2013: 697390 10.1155/2013/697390 . PMC:3657397. 23737843 PMC3657397

[3] Petelinc T, Polak T, Demšar L, Jamnik P. Fractionation of Phenolic Compounds Extracted from Propolis and Their Activity in the Yeast *Saccharomyces cerevisiae*. PLoS ONE. 2013; 8(2): e56104 10.1371/journal.pone.0056104 23409133 PMC3567072

[4] Teles F, Silva TM, Cruz Júnior FP, Honorato VH, Costa OH, Barbosa APF, et al Brazilian Red Propolis Attenuates Hypertension and Renal Damage in 5/6 Renal Ablation Model. PLoS ONE. 2015; 10: e0116535 10.1371/journal.pone.0116535 25607548 PMC4301812

[5] Bankova VS, Castro SLD, Marcucci MC. Propolis: recent advances in chemistry and plant origin. Apidologie. 2000; 31: 3–15. 10.1051/apido:2000102

[6] Park YK, Alencar SM, Aguiar CL. Botanical origin and chemical composition of Brazilian propolis. J. Agric. Food Chem. 2002; 50(9): 2502–2506. 10.1021/jf011432b 11958612

[7] Hata T, Tazawa S, Ohta S, Rhyu MR, Misaka T, Ichihara K. Artepillin C, a Major Ingredient of Brazilian Propolis, Induces a Pungent Taste by Activating TRPA1 Channels. PLoS ONE. 2012; 7(11): e48072 10.1371/journal.pone.0048072 23133611 PMC3487895

[8] Bankova V. Chemical diversity of propolis and the problem of standardization. J Ethnopharmacol. 2005; 100(1–2): 114–117. 10.1016/j.jep.2005.05.004 . 15993016

[9] Bankova VS, Popova M. Propolis of Stingless Bees: a Promising Source of Biologically Active Compounds. Pharmacogn Rev. 2007; 1: 88–92. Available: http://www.phcogrev.com/showBackIssue.asp?issn=09737847;year=2007;volume=1;issue=1;month=January-June.

[10] Massaro FC, Brooks PR, Wallace HM, Nsengiyumva V, Narokai L, Russell FD. Effect of Australian Propolis from Stingless Bees (*Tetragonula carbonaria*) on Pre-Contracted Human and Porcine Isolated Arteries. PLoS ONE. 2013; 8(11): e81297 10.1371/journal.pone.0081297 24260567 PMC3829943

[11] Castaldo S, Capasso F. Propolis, an old remedy used in modern medicine. Fitoterapia. 2002; 73: S1–S6. 10.1016/S0367-326X(02)00185-5 12495704

[12] Funakoshi-Tago M, Okamoto K, Izumi R, Tago K, Yanagisawa K, Narukawa Y, et al Anti-inflammatory activity of flavonoids in Nepalese propolis is attributed to inhibition of the IL-33 signaling pathway. Int. Immunopharmacol. 2015; 25: 189–198. 10.1016/j.intimp.2015.01.012 25614224

[13] Teixeira EW, Negri G, Meira RMSA, Message D, Salatino A. Plant origin of green propolis: bee behavior, plant anatomy and chemistry. Evid Based Complement Alternat Med. 2005; 2: 85–92. 10.1093/ecam/neh055 15841282 PMC1062148

[14] Piantino CR, Aquino FWB, Follegatti-Romero LA, Cabral FA. Supercritical CO_2_ extraction of phenolic compounds from *Baccharis dracunculifolia*. J. Supercrit Fluids. 2008; 47(2): 209–214. 10.1016/j.supflu.2008.07.012

[15] Daugsch A. The red propolis of northeast Brazil and its chemical and biological characteristics. D.Sc. Thesis, State University of Campinas. 2007. Available: http://www.bibliotecadigital.unicamp.br/document/?code=vtls000406573.

[16] Silva BB, Rosalen PL, Cury JA, Ikegaki M, Souza VC, Esteves A, et al Chemical composition and botanical origin of Red Propolis, a new type of Brazilian propolis. Evid Based Complement Alternat Med. 2008; 5(3): 313–316. 10.1093/ecam/nem059 . PMCid:PMC2529384. 18830449 PMC2529384

[17] Falcão SI, Vale N, Gomes P, Domingues MRM, Freire C, Cardoso SM, et al Phenolic profiling of Portuguese Propolis by LC–MS Spectrometry: Uncommon Propolis Rich in Flavonoid Glycosides. Phytochem Anal. 2013; 24(4): 309–318. 10.1002/pca.2412 23172843

[18] Alencar SM, Oldoni TLC, Castro ML, Cabral ISR, Costa Neto CM, Cury JA, et al Chemical composition and biological activity of a new type of Brazilian propolis: Red propolis. J Ethnopharmacol. 2007; 113(2): 278–283. 10.1016/j.jep.2007.06.005 . 17656055

[19] Barros MP, Sousa JPB, Bastos JK, Andrade SF. Effect of Brazilian green propolis on experimental gastric ulcers in rats. J Ethnopharmacol. 2007; 110(3): 567–571. 10.1016/j.jep.2006.10.022 . 17126509

[20] Ixtaina VY, Vega A, Nolasco SM, Tomás MC, Gimeno M, Bárzana E, et al Supercritical carbon dioxide extraction of oil from Mexican chia seed (*Salvia hispanica* L.): Characterization and process optimization. J Supercrit Fluids. 2010; 55: 192–199. 10.1016/j.supflu.2010.06.003

[21] Uribe JAR, Perez JIN, Kauil HC, Rubio GR, Alcocer CG. Extraction of oil from chia seeds with supercritical CO_2_. J Supercrit Fluids. 2011; 56(2): 174–178. 10.1016/j.supflu.2010.12.007

[22] Garmus TT, Paviani LC, Queiroga CL, Cabral FA. Extraction of phenolic compounds from pepper-rosmarin (*Lippia sidoides* Cham.) leaves by sequential extraction in fixed bed extractor using supercritical CO_2_, ethanol and water as solvents. J Supercrit Fluids. 2015; 99: 68–75. 10.1016/j.supflu.2015.01.016

[23] Pardo-Castaño C, Velásquez M, Bola-os G. Simple models for supercritical extraction of natural matter. J Supercrit Fluids. 2015; 97: 165–173. 10.1016/j.supflu.2014.09.044

[24] Biscaia D, Ferreira SRS. Propolis extracts obtained by low pressure methods and supercritical fluid extraction. J Supercrit Fluids. 2009; 51: 17–23. 10.1016/j.supflu.2009.07.011

[25] Chao-Rui C, Chun-Ting S, Jia-Jiuan W, Hsing-Ling Y, Shih-Lan H, Chieh-Ming JC. Precipitation of sub-micron particles of 3,5-diprenyl-4-hydroxycinnamic acid in Brazilian propolis from supercritical carbon dioxide anti-solvent solutions. J Supercrit Fluids. 2009; 50(2): 176–182. 10.1016/j.supflu.2009.06.001

[26] Machado BAS, Padilha FF, Nunes SB, Guedes CMC, Costa AS, Umsza-Guez MA, et al. Process for obtaining 3,5-diprenyl-4-hydroxycinnamic acid (Artepillin C) propolis extract using supercritical carbon dioxide and co-solvent. 2014. Brazilian patent BR1020140320121.

[27] Reverchon E, Marco I. Supercritical fluid extraction and fractionation of natural matter. J. Supercrit. Fluids. 2006; 38(2): 146–166. 10.1016/j.supflu.2006.03.020

[28] Mendiola JA, Herrero M, Cifuentes A, Ibáñes E. Use of compressed fluids for sample preparation: food applications. J Chromatogr. 2007; 1152(1–2): 234–246. 10.1016/j.chroma.2007.02.046 17353022

[29] Mattea F, Martín A, Cocero MJ. Carotenoid processing with supercritical fluids. J Food Eng. 2009; 93(3): 255–265. 10.1016/j.jfoodeng.2009.01.030

[30] Machado BAS, Pereira CG, Nunes SB, Padilha FF, Umsza-Guez MA. Supercritical Fluid Extraction Using CO_2_: Main Applications and Future Perspectives. Sep Sci Technol. 2013; 48(18): 2741–2760. 10.1080/01496395.2013.811422

[31] Wang BJ, Lien YH, Yu ZR. Supercritical fluid extractive fractionation-study of the antioxidant activities of propolis. Food Chem. 2004; 86(2): 237–243. 10.1016/j.foodchem.2003.09.031

[32] Frozza COS, Garcia CSC, Gambato G, Souza MDO, Salvador M, Moura S, et al Chemical characterization, antioxidant and cytotoxic activities of Brazilian red propolis. Food Chem. Toxicol. 2013; 52: 137–142. 10.1016/j.fct.2012.11.013 . 23174518

[33] Ngatu NR, Saruta T, Hirota R, Eitoku M, Muzembo BA, Matsui T, et al Antifungal efficacy of Brazilian green propolis extracts and honey on *Tinea capitis* and *Tinea versicolor*. Eur J Integr Med. 2011; 3(4): e281–e287. 10.1016/j.eujim.2011.10.001

[34] Fischer G, Conceição FR, Leite FPL, Dummer LA, Vargas GD'A, Hübner SO, et al Immunomodulation produced by a green propolis extract on humoral and cellular responses of mice immunized with SuHV-1. Vaccine. 2007; 25(7): 1250–1256. 10.1016/j.vaccine.2006.10.005 . 17084001

[35] Cheung KW, Sze DM, Chan WK, Deng RX, Tu W, Chan GCF. Brazilian green propolis and its constituent, Artepillin C inhibits allogeneic activated human CD4 T cells expansion and activation. J Ethnopharmacol. 2011; 138(2): 463–471. 10.1016/j.jep.2011.09.031 . 21964192

[36] Barros MP, Lemos M, Maistro EL, Leite MF, Sousa JPB, Bastos JK, et al Evaluation of antiulcer activity of the main phenolic acids found in Brazilian Green. J Ethnopharmacol. 2008; 120(3): 372–377. 10.1016/j.jep.2008.09.015 . 18930797

[37] Paulino N, Abreu SRL, Uto Y, Koyama D, Nagasawa H, Hori H, et al Anti-inflammatory effects of a bioavailable compound, Artepillin C, in Brazilian propolis. Eur. J. Pharmacol. 2008; 587(1–3): 296–301. 10.1016/j.ejphar.2008.02.067 . 18474366

[38] Fernandes FH, Guterres ZR, Garcez WS, Lopes SM, Corsino J, Garcez. FR Assessment of the (anti)genotoxicity of brown propolis extracts from Brazilian Cerrado biome in a *Drosophila melanogaster* model. Food Res Int. 2014; 62: 20–26. 10.1016/j.foodres.2014.02.029

[39] Wilson MB, Brinkman D, Spivak M, Gardner G, Cohen JD. Regional variation in composition and antimicrobial activity of US propolis against *Paenibacillus larvae* and *Ascosphaera apis*. J. Invertebr. Pathol. 2015; 124: 44–50. 10.1016/j.jip.2014.10.005 . 25450740

[40] AOAC. Association of Official Analytical Chemists. Official methods of analysis of AOAC International. 16th ed. Washington: AOAC International; 1997.

[41] Blight EG, Dyer WJ. A Rapid Method of Total Lipid Extraction and Purification. Can J Biochem Physiol. 1959; 37(8): 911–917. . 13671378 10.1139/o59-099

[42] Van-Soest PJ, Wine RH. Use of detergents in analysis of fibrous feeds. In: Determination of plant cell wall constituents. J Assoc Off Anal Chem. 1967; 50: 50 Available: http://catalogo.latu.org.uy/doc_num.php?explnum_id=1418.

[43] Machado BAS, Barreto GA, Costa AS, Costa SS, Silva RPD, Silva DF, et al Determination of parameters for the supercritical extraction of antioxidant compounds from green propolis using carbon dioxide and ethanol as co-solvent. PLoS ONE. 2015; 10(8): e0134489 10.1371/journal.pone.0134489 26252491 PMC4529176

[44] Brazil. Ministry of Health. National Health Surveillance Agency (ANVISA). Resolution n° 899, of may 29, 2003: Guide to the validation of analytical and bioanalytical methods. Available: http://portal.anvisa.gov.br/wps/wcm/connect/4983b0004745975da005f43fbc4c6735/RE_899_2003_Determina+a+publica%C3%A7%C3%A3o+do+Guia+para+valida%C3%A7%C3%A3o+de+m%C3%A9todos+anal%C3%ADticos+e+bioanal%C3%ADticos.pdf?MOD=AJPERES.

[45] INMETRO. National Institute of Metrology, Standardization and Industrial Quality. Guidelines for Chemical Testing Methods Validation. 2011. Available: http://www.inmetro.gov.br/Sidoq/Arquivos/Cgcre/DOQ/DOQ-Cgcre-8_04.pdf.

[46] Singleton VL, Rossi JA. Colorimetry of total phenolics with phosphomolybdic-phosphotungstic acid reagents. Am J Enol Vitic. 1965; 16(3): 144–158. Available: http://ajevonline.org/content/16/3/144.

[47] Singleton VL, Orthofer R, Lamuela-Raventos RM. Analysis of total phenols and other oxidation substrates and antioxidants by means of FolinCiocalteu reagent. Meth Enzymol. 1999; 299: 152–178. 10.1016/S0076-6879(99)99017-1

[48] Peschel W, Sánchez-Rabaneda F, Diekmann W, Plescher A, Gartzia I, Jiménez D, et al An industrial approach in the search of natural antioxidants from vegetable and fruit wastes. Food Chem. 2006; 97(1):137–150. 10.1016/j.foodchem.2005.03.033

[49] Marcucci MC, Ferreres F, Guarcía-Viguera C, Bankova VS, Castro SL, Dantas AP, et al Phenolic compounds from Brazilian propolis with pharmacological activities. J Ethnopharmacol. 2001; 74(2): 105–112. 10.1016/S0378-8741(00)00326-3 . 11167028

[50] Brand-William W, Cuvelier ME, Berset C. Use of free radical method to evaluate antioxidant activity. Lebenson Wiss Technol. 1995; 28: 25–30. 10.1016/S0023-6438(95)80008-5

[51] Molyneux P. The use of the stable free radical diphenylpicrylhydrazyl (DPPH) for estimating antioxidant activity. Songklanakarin J Sci Technol. 2004; 26: 211–219. Available: http://rdo.psu.ac.th/sjstweb/journal/26-2/07-DPPH.pdf.

[52] Van-Den-Berg R, Haenen GRMM, Van-Den-Berg H, Bast A. Applicability of an improved Trolox equivalent antioxidant capacity (TEAC) assay for evaluation of antioxidant capacity measurements of mixtures. Food Chem. 1999; 66(4): 511–517. 10.1016/S0308-8146(99)00089-8

[53] Kim DO, Chun OK, Kim YJ, Moon HY, Lee CY. Quantification of polyphenolics and their antioxidant capacity in fresh plums. J Agric Food Chem. 2003; 51(22): 6509–6515. 10.1021/jf0343074 . 14558771

[54] NCCLS. National Committee for Clinical Laboratory Standards. Methods for dilution antimicrobial susceptibility tests for bacteria that grow aerobically (M100-S10 (M7)). Approved standard. 5ªed. Wayne, PA: NCCLS; 2000.

[55] Koo H, Rosalen PL, Cury JA, Ambrosano GMB, Murata RM, Yatsuda R, et al Effect of a New Variety of *Apis mellifera* Propolis on Mutants Streptococci. Curr Microbiol. 2000; 41(3): 192–196. 10.1007/s0028400101170 . 10915206

[56] Novak EM, Silva MSC, Marcucci MC, Sawaya ACHF, López BGC, Fortes MAZ, et al Antitumoural activity of Brazilian red propolis fraction enriched with xanthochymol and formononetin: An *in vitro* and in vivo study. J Funct Foods. 2014; 11: 91–102. 10.1016/j.jff.2014.09.008

[57] Busatti HG, Gomes MA. A Simple Colourimetric Method to Determine AntiGiardial Activity of Drugs. Parasitol Res. 2007; 101(3): 819–821. 10.1007/s00436-007-0525-8 . 17387517

[58] Ozcan M, Ayar A. Effect of propolis extracts on butter stability. J Food Qual. 2003; 26: 65–73. 10.1111/j.1745-4557.2003.tb00227.x

[59] Narbona E, García-García E, Vazquez-Araújo L, Carbonell-Barrachina AA. Volatile composition of functional ‘*a la Piedra*’*turrón* with propolis. Int J Food Sci Technol. 2010; 45(3): 569–577. 10.1111/j.1365-2621.2009.02167.x

[60] Ali FH, Kassem GM, Atta-Alla OA. Propolis as a natural decontaminant and antioxidant in fresh oriental sausage. Vet Ital. 2010; 46(2): 167–172. . 20560126

[61] Brazil. Ministry of Health. National Health Surveillance Agency (ANVISA). Normative Instruction n° 3, of January 19, 2001: Technical Regulations of identify and Quality of bee venom, royal Bee, Jelly Wax, Lyophilized Royal Jelly, Bee Pollen, Propolis and Propolis Extract. Available: http://extranet.agricultura.gov.br/sislegis-consulta/consultarLegislacao.do?operacao=visualizar&id=1798.

[62] Bankova VS, Popova SS, Marekov NL, Maksimova V. The chemical composition of propolis fractions with antiviral action. Acta Microbiol Bulg. 1988; 23: 52–57. . 3247872

[63] Marcucci MC, Rodriguez J, Ferrerez F, Bankova V, Groto R, Popov S. Chemical composition of Brazilian Propolis from São Paulo State. Z Naturforsch, C, Biosci. 1998; 53c: 117–119. Available: http://zfn.mpdl.mpg.de/data/Reihe_C/53/ZNC-1998-53c-0117.pdf.

[64] Dias LG, Pereira AP, Estevinho LM. Comparative study of different Portuguese samples of propolis: Pollinic, sensorial, physicochemical, microbiological characterization and antibacterial activity. Food Chem Toxicol. 2012; 50(12): 4246–4253. 10.1016/j.fct.2012.08.056 22981908

[65] Moreira TF. Chemical composition of propolis: vitamins and aminoacids. Braz Rev Farmacogn. 1986; 1(1): 12–19. 10.1590/S0102-695X1986000100003

[66] Bonvehi JS, Coll FV, Jordà RE. The composition, active components and bacteriostatic activity of propolis in dietetics. J Am Oil Chem Soc. 1994; 71(5): 529–532. 10.1007/BF02540666

[67] Funari CS, Ferro VO. Propolis analysis. Braz J Food Technol. 2006; 26: 171–178. 10.1590/S0101-20612006000100028

[68] Attia YA, Al-Hamid AEA, Ibrahim MS, Al-Harthi MA, Bovera F, Elnaggar ASh. Productive performance, biochemical and hematological traits of broiler chickens supplemented with propolis, bee pollen, and mannan oligosaccharides continuously or intermittently. Livest Sci. 2014; 164: 87–95. 10.1016/j.livsci.2014.03.005

[69] Tylkowski B, Trusheva B, Bankova V, Giamberini M, Peev G, Nikolova A. Extraction of biologically active compounds from propolis and concentration of extract by nanofiltration. J Memb Sci. 2010; 348(1–2): 124–130. 10.1016/j.memsci.2009.10.049

[70] Budel JM, Duarte MR, Santos CAM, Farago PV. Leaf and stem morpho-anatomy of *Baccharis dracunculifolia* DC., Asteraceae. Acta Farm Bonaerense. 2004; 23(4): 477–483. Available: http://www.latamjpharm.org/trabajos/23/4/LAJOP_23_4_1_8_I54P2Y1561.pdf.

[71] Budel JM, Duarte MR. Pharmacobotanical study of aerial vegetative parts of *Baccharis anomala* DC., Asteraceae. Braz Rev Farmacogn. 2008; 18: 761–768. 10.1590/S0102-695X2008000500022

[72] Bastos EMAF, Santana RA, Calaça-Costa AGF, Thiago PS. Interaction between *Apis mellifera* L. and *Baccharis dracunculifolia* DC, that favours green propolis production in Minas Gerais. Braz J Biol. 2011; 71(3): 727–734. 10.1590/S1519-69842011000400018 21881797

[73] Sawaya ACHF, Cunha IBS, Marcucci MC, Rodrigues RFD, Eberlin MN. Brazilian propolis of *Tetragonisca angustula* and *Apis mellifera*. Apidologie. 2006; 37(3): 398–407. 10.1051/apido:2006011

[74] Lotti C, Fernandez MC, Piccinelli AL, Cuesta-Rubio O, Hernandez IM, Rastrelli L. Chemical constituents of red Mexican propolis. J Agric Food Chem. 2010; 58(4): 2209–2213. 10.1021/jf100070w . 20121106

[75] Costa SS, Machado BAS, Souza CO, Druzian JI, Guimarães AG. Bi-Functional biobased packing of the cassava starch, glycerol, licuri nanocellulose and red propolis. Plos One. 2014; 9(11): e112554–e112554. 10.1371/journal.pone.0112554 . PMCid:PMC4226550. 25383783 PMC4226550

[76] Begnini KR, Leon PMM, Thurow H, Schultze E, Campos V, Rodrigues FM, et al Brazilian Red Propolis Induces Apoptosis-Like Cell Death and Decreases Migration Potential in Bladder Cancer Cells. Evid Based Complement Alternat Med. 2014; 2014: 1–13 10.1155/2014/639856 . PMCid:PMC4235187. 25530785 PMC4235187

[77] Cottica SM, Sabik H, Antoine C, Fortin J, Graveline N, Visentainer JV, et al Characterization of Canadian propolis fractions obtained from two-step sequential extraction. Lebenson Wiss Technol. 2015; 60: 609–614. 10.1016/j.lwt.2014.08.045

[78] Christov R, Trusheva B, Popova M, Bankova V, Bertrand M. Chemical composition of propolis from Canada, its antiradical activity and plant origin. Nat Prod Res. 2005; 19:673–678. 10.1080/14786410512331328159 16076637

[79] Nagai T, Inoue R, Inoue H, Suzuki N. Preparation and antioxidant properties of water extract of propolis. Food Chem. 2003; 80: 29–33. 10.1016/S0308-8146(02)00231-5

[80] Valencia D, Alday E, Robles-Zepeda R, Garibay-Escobar A, Galvez-Ruiz JC, Salas-Reyes M, et al Seasonal effect on chemical composition and biological activities of Sonoran propolis. Food Chem. 2012; 131(2): 645–651. 10.1016/j.foodchem.2011.08.086

[81] Bankova V, Popova M, Trusheva B. Propolis volatile compounds: chemical diversity and biological activity: a review. Chem Cent J. 2014; 8:28 10.1186/1752-153X-8-28 24812573 PMC4014088

[82] Chaillou LL, Nazareno MA. Bioactivity of propolis from Santiago del Estero, Argentina, related to their chemical composition. Lebenson Wiss Technol. 2009; 42(8): 1422–1427. 10.1016/j.lwt.2009.03.002

[83] Kumazawa S, Hamasaka T, Nakayama T. Antioxidant activity of propolis of various geographic origins. Food Chem. 2004; 84(3): 329–339. 10.1016/S0308-8146(03)00216-4

[84] Kalogeropoulos N, Konteles SJ, Troullidou E, Mourtzinos I, Karathanos VT. Chemical composition, antioxidant activity and antimicrobial properties of propolis extracts from Greece and Cyprus. Food Chem. 2009; 116(2): 452–461. 10.1016/j.foodchem.2009.02.060

[85] Choi YM, Noh DO, Cho SY, Suh HJ, Kim KM, Kim JM. Antioxidant and antimicrobial activities of propolis from several regions of Korea. Lebenson Wiss Technol. 2006; 39(7): 756–761. 10.1016/j.lwt.2005.05.015

[86] Miguel MG, Nunes S, Dandlen SA, Cavaco AM, Antunes MD. Phenols and antioxidant activity of hydro-alcoholic extracts of propolis from Algarve, South of Portugal. Food Chem. Toxicol. 2010; 48: 3418–3423. 10.1016/j.fct.2010.09.014 . 20849908

[87] Zordi N, Cortesi A, Kikic I, Moneghini M, Solinas D, Innocenti G, et al The supercritical carbon dioxide extraction of polyphenols from propolis: a central composite design approach. J Supercrit Fluids. 2014; 95: 491–498. 10.1016/j.supflu.2014.10.006

[88] Laskar RA, Sk I, Roy N, Begum NA. Antioxidant activity of Indian propolis and its chemical constituents. Food Chem. 2010; 122: 233–237. 10.1016/j.foodchem.2010.02.068

[89] Kimoto T, Arai S, Kohguchi M, Aga M, Nomura Y, Micallef MJ, et al Apoptosis and suppression of tumor growth by artepillin C extracted from Brazilian propolis. Cancer Detect Prev. 1998; 22(6): 506–515. 10.1046/j.1525-1500.1998.00020.x . 9824373

[90] Chen CR, Lee YN, Lee MR, Chang CMJ. Supercritical fluids extraction of cinnamic acid derivatives from Brazilian propolis and the effect on growth inhibition of colon cancer cells. J Taiwan Inst Chem Eng. 2009; 40(2): 130–135. 10.1016/j.jtice.2008.07.014

[91] Ikeda R, Yanagisawa M, Takahashi N, Kawada T, Kumazawa S, Yamaotsu N, et al Brazilian propolis-derived components inhibit TNF-α-mediated downregulation of adiponectin expression via different mechanisms in 3T3-L1 adipocyte. Biochim Biophys Acta—General Subjects. 2011; 1810(7): 695–703. 10.1016/j.bbagen.2011.04.007 . 21554928

[92] Tazawa S, Warashina T, Noro T. On the chemical evaluation of propolis. J Nat Med (in Japanese). 2000; 54: 306–313. Available: http://chemport.cas.org/cgi-bin/sdcgi?APP=ftslink&action=reflink&origin=jstage2&version=1.0&coi=1%3ACAS%3A528%3ADC%252BD3MXhvVCjsLY%253D&md5=facc10806e7bfb2f89bd486b9076b58c.

[93] Shimizu K, H Ashida H, Matsuura Y, Kanazawa K. Antioxidative bioavailability of Artepillin C in Brazilian propolis. Arch. Biochem. Biophys. 2004; 424(2): 181–188. 10.1016/j.abb.2004.02.021 15047190

[94] Ahn MR, Kumazawa S, Usui Y, Nakamura J, Matsuka M, Zhu F, et al Antioxidant activity and constituents of propolis collected in various areas of China. Food Chem. 2007; 101:1383–1392.

[95] Chen CR, Shen CT, Wu JJ, Yang HL, Hsu SL, Chang CMJ. Precipitation of sub-micron particles of 3,5-diprenyl-4-hydroxycinnamic acid in Brazilian propolis from supercritical carbon dioxide anti-solvent solutions. J Supercrit Fluids. 2009; 50(2): 176–182. 10.1016/j.supflu.2009.06.001

[96] Donelian A, Carlson LHC, Lopes TJ, Machado RAF. Comparison of extraction of patchouli (Pogostemon cablin) essential oil with supercritical CO_2_ and by steam distillation. J Supercrit Fluids. 2009; 48: 15–20. 10.1016/j.supflu.2008.09.020

[97] Glisic S, Ivanovic J, Ristic M, Skala D. Extraction of sage (*Salvia officinalis* L.) by supercritical CO_2_: Kinetic data, chemical composition and selectivity of diterpenes. J Supercrit Fluids. 2010; 52: 62–70. 10.1016/j.supflu.2009.11.009

[98] Vargas CE, Mendes MF, Azevedo DA, Pessoa FLP, Uller AC. Extraction of the essential oil of abajeru (Chrysobalanus icaco) using supercritical CO_2_. J Supercrit Fluids. 2010; 54(2): 171–177. 10.1016/j.supflu.2009.12.007

[99] Zhao S, Zhang D. Supercritical CO_2_ extraction of Eucalyptus leaves oil and comparison with Soxhlet extraction and hydro-distillation methods. Sep Purif Technol. 2014; 133: 443–451. 10.1016/j.seppur.2014.07.018

[100] Sun Y, Liu Z, Wang J, Tian W, Zhou H, Zhu L, et al Supercritical fluid extraction of paeonol from *Cynanchum paniculatum* (Bge.) Kitag. and subsequent isolation by high-speed counter-current chromatography coupled with high-performance liquid chromatography-photodiode array detector. Sep Purif Technol. 2008; 64(2): 221–226. 10.1016/j.seppur.2008.10.007

[101] Wu J, Luyu Q. Supercritical multiple extraction of bee glue. 2000. Chinese Patent, CN1258511.

[102] Kimoto N, Hirose M, Kawabe M, Satoh T, Miyataka H, Shirai T. Post-initiation effects of a super critical extract of propolis in a rat two-stage carcinogenesis model in female F344 rats. Câncer Lett. 1999; 147(1–2): 221–227. 10.1016/S0304-3835(99)00305-5 10660110

[103] Koru O, Toksoy F, Acikel CH, Tunca YM, Baysallar M, Guclu AU, et al *In vitro* antimicrobial activity of propolis samples from different geographical origins against certain oral pathogens. Anaerobe. 2007; 13(3–4): 140–145. 10.1016/j.anaerobe.2007.02.001 . 17475517

[104] Vardar-Ünlü G, Silici S, Ünlü M. Composition and *in vitro* antimicrobial activity of Populus buds and poplar-type propolis. World J Microbiol Biotechnol. 2008; 24(7): 1011–1017. 10.1007/s11274-007-9566-5

[105] Kim YH, Chung HJ. The effects of Korean Propolis against foodborne pathogens and transmission electron microscopic examination. N Biotechnol. 2011; 28(6): 713–718. 10.1016/j.nbt.2010.12.006 . 21232643

[106] Silva JC, Rodrigues S, Feás X, Estevinho LM. Antimicrobial activity, phenolic profile and role in the inflammation of propolis. Food Chem Toxicol. 2012; 50(5): 1790–1795. 10.1016/j.fct.2012.02.097 22425940

[107] Silici S, Kutluca S. Chemical composition and antibacterial activity of propolis collected by three different races of honeybees in the same region. J Ethnopharmacol. 2005; 99: 69–73. 10.1016/j.jep.2005.01.046 . 15848022

[108] Mohammadzadeh S, Shariatpanahi M, Hamedi M, Ahmadkhaniha R, Samadi N, Ostad SN. Chemical composition, oral toxicity and antimicrobial activity of Iranian propolis. Food Chem. 2007; 103(4): 1097–1103. 10.1016/j.foodchem.2006.10.006

[109] Popova MP, Bankova VS, Bogdanov S, Tsvetkova I, Naydenski C, Marcazzan GL, et al Chemical characteristics of poplar type propolis of different geographic origin. Apidologie. 2007; 38(3): 306–311. 10.1051/apido:2007013

[110] Jug M, Končić MZ, Kosalec I. Modulation of antioxidant, chelating and antimicrobial activity of poplar chemo-type propolis by extraction procures. Lebenson Wiss Technol. 2014; 57(2): 530–537. 10.1016/j.lwt.2014.02.006

[111] Hayacibara MF, Koo H, Rosalen PL, Duarte S, Franco EM, Bowen WH, et al *In vitro* and in vivo effects of isolated fractions of Brazilian propolis on caries development. J Ethnopharmacol. 2005; 101(1–3): 110–115. 10.1016/j.jep.2005.04.001 . 15913934

[112] Hegazi AG, Abd El Hady FK. Egyptian propolis: 3. Antioxidant, antimicrobial activities and chemical composition of propolis from reclaimed lands. Z Naturforsch C. 2002; 57(3–4): 395–402. 10.1515/znc-2002-3-432 . 12064746

[113] Ghasem YB, Ownagh A, Hasanloei M. Antibacterial and antifungal activity of Iranian propolis against Staphylococcus aureus and Candida albicans. Pak J Biol Sci. 2007; 10(8): 1343–1345. 10.3923/pjbs.2007.1343.1345 . 19069941

[114] Patel J, Ketkar S, Patil S, Fearnley J, Mahadik KR, Paradkar AR. Potentiating antimicrobial efficacy of propolis through niosomal-based system for administration. Integr Med Res. 2014 10.1016/j.imr.2014.10.004 PMC5481798 28664114

[115] Sawaya ACHF, Souza KS, Marcucci MC, Cunha IBS, Shimizu MT. Analysis of the composition of Brazilian propolis extracts by chromatography and evaluation of their *in vitro* activity against gram-positive bacteria. Braz J Microbiol. 2004; 35(1–2): 104–109. 10.1590/S1517-83822004000100017

[116] Melliou E, Stratis E, Chinou I. Volatile constituents of propolis from various regions of Greece–Antimicrobial activity. Food Chem. 2007; 103(2): 375–380. 10.1016/j.foodchem.2006.07.033

[117] Bankova V, Boudourova-Krasteva G, Popov S, Sforcin JM, Funari SRC. Seasonal variations of the chemical composition of Brazilian propolis. Apidologie. 1998; 29(4): 361–367. 10.1051/apido:19980406

[118] Takaisi-Kikuni NB, Schilcher H. Electron microscopic and microcalorimetric investigations of the possible mechanism of the antibacterial action of a defined propolis provenance. Planta Med. 1994; 60(3): 222–227. 10.1055/s-2006-959463 . 8073087

[119] Tsuchiya H, Iinuma M. Reduction of membrane fluidity by antibacterial sophoraflavone G isolated from Sophora exigua. Phytomedicine. 2000; 7(2): 161–165. 10.1016/S0944-7113(00)80089-6 10839220

[120] Buriol L, Finger D, Schmidt EM, Santos JMT, Rosa MR, Quináia SP, et al Composição química e atividade biológica de extrato oleoso de própolis: uma alternativa ao extrato etanólico. Quím Nova. 2009; 32(2): 296–302. 10.1590/S0100-40422009000200006

[121] Franchi GC Jr, Moraes CS, Toreti VC, Daugsch A, Nowill AE, Park YK. Comparison of Effects of the Ethanolic Extracts of Brazilian Propolis on Human Leukemic Cells As Assessed with the MTT Assay. J Evid Based Complementary Altern Med. 2012; 2012: 1–6. 10.1155/2012/918956 PMC3182072 21966298

[122] Popolo A, Piccinelli LA, Morello S, Cuesta-Rubio O, Sorrentino R, Rastrelli L, et al Antiproliferative activity of brown Cuban propolis extract on human breast cancer cells. Nat Prod Comun. 2009; 4(12):1711–1716. . 20120113

[123] Piccinelli AL, Lotti C, Campone L, Cuesta-Rubio O, Campo Fernandez M, Rastrelli L. Cuban and Brazilian red propolis: botanical origin and comparative analysis by high-performance liquid chromatography-photodiode array detection/electrospray ionization tandem mass spectrometry. J Agric Food Chem. 2011; 22;59(12):6484–91. 10.1021/jf201280z . 21598949

[124] Frozza COS, Ribeiro TS, Gambato G, Menti C, Moura S, Pinto PM, et al Proteomic analysis identifies differentially expressed proteins after red propolis treatment in Hep-2 cells. Food Chem. Toxicol. 2014; 63: 195–204. 10.1016/j.fct.2013.11.003 . 24239894

[125] López BG, Schmidt EM, Eberlin MN, Sawaya AC. Phytochemical markers of different types of red propolis. Food Chem. 2014; 1(146):174–180. 10.1016/j.foodchem.2013.09.063 24176329

[126] Cuesta-Rubio O, Fontana-Uriba BA, Ramirez-Apan T, Cardenas J. Polyisoprenylated benzophenones in Cuban propolis: biological activity of nemorosone. Z Naturforsch. 2002; 57:372–378. 10.1515/znc-2002-3-429 12064743

[127] Ye Y, Hou R, Chen J, Mo L, Zhang J, Huang Y, et al Formononetin-induced Apoptosis of Human Prostate Cancer Cells Through ERK1/2 Mitogen-activated Protein Kinase Inactivation. Horm Metab Res. 2012; 44(04): 263–267. 10.1055/s-0032-1301922 22328166

[128] Zhang X, Bi L, Ye Y, Chen J. Formononetin Induces Apoptosis in PC-3 Prostate Cancer Cells Through Enhancing the Bax/Bcl-2 Ratios and Regulating the p38/Akt Pathway. Nutr Cancer. 2014; 66(4): 656–661. 10.1080/01635581.2014.894098 24666255

[129] Matsumoto K, Akao Y, Kobayashi E, Ito T, Ohguchi K, Tanaka T, et al Cytotoxic benzophenone derivatives from *Garcinia* species display a strong apoptosis-inducing effect against human leukaemia cell lines. Biol. Pharm. Bull. 2003; 26(4): 569–571. 10.1248/bpb.26.569 . 12673047

[130] Chen J, Sun L. Formononetin-induced apoptosis by activation of Ras/p38 mitogen-activated protein kinase in estrogen receptor-positive human breast cancer cells. Horm Metab Res. 2012; 44(13): 943–948. 10.1055/s-0032-1321818 . 22828872

[131] Shimizu K, Ashida H, Matsuura Y, Kanazawa K. Antioxidative bioavailability of artepillin C in Brazilian propolis. Arch Biochem Biophys. 2004; 424(2): 181–188. 10.1016/j.abb.2004.02.021 15047190

[132] Carvalho AA, Finger D, Machado CS, Schmidt EM, Costa PM, Alves APNN, et al In vivo antitumoural activity and composition of an oil extract of Brazilian propolis. Food Chem. 2011; 126(3): 1239–1245. 10.1016/j.foodchem.2010.12.035

[133] Kimoto T, Aga M, Hino K, Koya-Miyata S, Yamamoto Y, Micallef MJ, et al Apoptosis of human leukemia cells induced by Artepillin C, an active ingredient of Brazilian propolis. Anticancer Res. 2001; 21(1A): 221–228. . 11299738

[134] Matsuno T, Jung SK, Matsumoto Y, Saito M, Morikawa J. Preferential cytotoxicity to tumor cells of 3,5-diprenyl-4-hydroxycinnamic acid (Artepillin C) isolated from própolis. Anticancer. 1997; 17: 3565–3568. . 9413203

[135] Kimoto T, Koya-Miyata S, Hino K, Micallef MJ, Hanaya T, Arai S, et al Pulmonary carcinogenesis induced by ferric nitrilotriacetate in mice and protection from it by Brazilian propolis and artepillin C. Virchows Arch. 2001; 438(3): 259–270. 10.1007/s004280000350 . 11315623

[136] Akao Y, Maruyama H, Matsumoto K, Ohguchi K, Nishizawa K, Sakamoto T. Cell growth inhibitory effect of cinnamic acid derivatives from propolis on human tumor cell lines. Biol. Pharm. Bull. 2003; 26(7): 1057–1059. 10.1248/bpb.26.1057 12843641

